# Taxonomic re-identification and biotechnological potential of a Brazilian *Aspergillus* section *Flavi* collection: exploring kojic acid production

**DOI:** 10.1007/s11274-026-05137-z

**Published:** 2026-07-18

**Authors:** Maria Tamara de Caldas Felipe, Renan do Nascimento Barbosa, Jadson Diogo Pereira Bezerra, Rafael Barros de Souza, Walter de Paula Pinto Neto, Rayssa Karla Silva, Cristina Maria de Souza-Motta

**Affiliations:** 1 Departamento de Micologia Prof. Chaves Batista, Universidade Federal de Pernambuco (UFPE), Av. Prof. Moraes Rego, s/n, Centro de Biociências, Cidade Universitária, Recife, 50670-901 Pernambuco Brazil; 2 Laboratório de Micologia, Departamento de Biociências e Tecnologia, Instituto de Patologia Tropical e Saúde Pública (IPTSP), Universidade Federal de Goiás (UFG), Rua 235, s/n, Setor Universitário, Goiânia, 74605-050 Goiás Brazil; 3 Laboratório de Metabolismo Microbiano, Instituto de Ciências Biológicas, Universidade de Pernambuco (UPE), s/n, Santo Amaro, Recife, 50100-130 Pernambuco Brazil

**Keywords:** 5-hydroxy-2-hydroxymetyl-4-pyrone, Culture collection, Fungal phylogeny, Sugarcane molasses, Fungal taxonomy

## Abstract

Species of *Aspergillus* section *Flavi* are recognized for their ability to produce metabolites, such as mycotoxins and organic acids. Kojic acid has gained widespread recognition in the cosmetics market for its efficacy in lightening skin blemishes. The importance of these species makes their correct identification and preservation essential. This study aimed to re-identify, taxonomically, *Aspergillus* section *Flavi* strains stored at the URM culture collection (WDCM 604) in Brazil and to investigate their potential for kojic acid production. Initially, 194 *Aspergillus* section *Flavi* strains were reactivated and authenticated based on DNA sequences (*CaM* and *BenA*). Furthermore, 99 strains were evaluated on synthetic solid medium to verify their ability to produce kojic acid. A representative strain from each species was selected to evaluate kojic acid production via submerged fermentation, using sugarcane molasses and corn steep liquor as carbon and nitrogen sources, respectively. Quantitative analysis of kojic acid was performed by high-performance liquid chromatography. In submerged fermentation, *Aspergillus caelatus* URM 4242 showed the highest production (10.65 ± 0.4 g/L with a yield of 0.54 g/g). Our results highlight the importance of culture collections, taxonomic re-identification, and assessment of the biotechnological potential of strains deposited in these biobanks.

**Supplementary Information:**

The online version contains supplementary material available at 10.1007/s11274-026-05137-z.

## Introduction

*Aspergillus* section *Flavi* encompasses species with globose conidia, varying from rough to echinulate, and exhibiting yellow-green to brown shades conidia *en masse* and dark-colored sclerotia (Norlia et al. 2019). The species in this section are cosmopolitan, with a wide distribution worldwide (Perrone et al. 2014). The best-known representative of this section is *A. flavus* (Varga et al. 2011; Norlia et al. 2019). Currently, 35 species are accepted within the section, each with distinct ecological and morphological characteristics (Wang et al. 2021; Silva et al. 2022a, b; Visagie et al. 2024). Some representatives are also found as pathogens of humans and other animals (Hedayati et al. 2007). However, they are mainly reported in grains of global economic interest, causing contamination and losses (Varga et al. 2011; Frisvad et al. 2019).

Section *Flavi* species exhibit both beneficial and harmful traits. Several species are known to produce industrially valuable enzymes and metabolites, including those used in the fermentation of soy-based products such as soy sauce, miso, and sake (Raper and Fennell 1969; Samson et al. 2010; Varga et al. 2011; Hong et al. 2013). On the other hand, they are also considered phytopathogens, causing plant infections, such as yellow mold in peanuts (Zakaria 2024), and are of great concern due to the production of highly toxic compounds (mycotoxins), such as aflatoxins (AFs), 3-nitropropionic acid, tenuazonic acid, cyclopiazonic acid (CPA) and aspergillic acid, which can contaminate grains and pose risks to human and other animals health (Samson et al. 2007, 2010; Rodrigues et al. 2009; Varga et al. 2011; Frisvad and Larsen 2015; Frisvad et al. 2019; Norlia et al. 2019; Kjærbølling et al. 2020; Visagie and Houbraken 2020). *Aspergillus flavus* also has biotechnological importance, as it can produce organic acids and enzymes, including kojic acid, a secondary metabolite produced by most species in section *Flavi*, except for *A. avenaceus* and *A. coremiiformis* (Frisvad et al. 2019). Kojic acid has a variety of potential applications, including antibiotic (Wu et al. 2019), antidiabetic (Wei et al. 2011), anti-inflammatory (Li et al. 2021) and skin-lightening effects (Noh et al. 2009).

The infrageneric classification of *Aspergillus* has evolved significantly over time, largely driven by technological advances and the adoption of the “one fungus = one name” principle. Early classification systems were primarily based on morphological characteristics, with the pioneering work of Thom and Church (1926), followed by Thom and Raper (1945), who introduced an informal subgeneric classification by grouping species into “groups.” Raper and Fennel (1969) further developed this structure and are considered the first to propose an infrageneric classification for the genus *Aspergillus*. Several other researchers have also contributed to the refinement of this taxonomy over the decades, expanding our understanding of the diversity and organization of the group. Peterson et al. (2001) studied the phylogenetic relationships among *Aspergillus* species using ribosomal RNA sequences and proposed three subgenera and 16 sections. More comprehensive studies subsequently subdivided the genus into six subgenera and 28 sections (Kocsubé et al. 2016; Houbraken et al. 2020; Visagie et al. 2024). Although subgeneric classifications have become more structured, species-level taxonomy within section *Flavi* remains challenging. As a result, the taxonomy of aflatoxigenic species in this section is still partially unresolved. To address these challenges, current identification of *Aspergillus* species relies on a polyphasic approach, mainly integrating morphological characteristics, multilocus DNA sequence data (e.g., partial β-tubulin – *BenA*, calmodulin – *CaM* and RNA polymerase II second largest subunit – *RPB2*) and, when possible, extrolite profiles (Frisvad et al. 2019; Houbraken et al. 2020; Visagie et al. 2024). These changes have led to the refinement of subgeneric boundaries and the reorganization of species into more natural groupings. Currently, species identification within *Aspergillus* relies on a polyphasic approach that combines morphological characteristics, extrolite profiles, and multilocus DNA sequence data (e.g., *BenA*, *CaM* and *RPB2*).

Fungal collections are important sources of microbiological resources that play a fundamental role in mycology, as they maintain viable strains with great potential for various sectors, including the pharmaceutical industry, medicine, environmental science, agriculture, education, and research (Souza et al. 2015; Bezerra et al. 2017). The URM culture collection (Micoteca URM), located at the Universidade Federal de Pernambuco (Recife, Brazil), stands out as one of Brazil’s most important fungal repositories. Established in 1954 by Prof. Augusto Chaves Batista, this collection has grown to house a remarkable diversity, encompassing approximately 9,000 fungal names and around 100,000 strains, including zygosporic fungi and species from *Ascomycota* and *Basidiomycota* (Felipe et al. 2024). The URM culture collection is affiliated with the World Federation of Cultural Collections (WFCC) and registered under number WDCM 604 (Souza-Motta and Barbosa 2022; Felipe et al. 2024; Ccinfo 2024).

Several *Aspergillus* section *Flavi* strains deposited in the URM culture collection were originally identified only based on morphological characters, using dichotomous keys available at the time (i.e., Raper and Fennell 1969; Thom and Church 1926; Thom and Raper 1945; Samson 1979; Samson and Pitt 1986, 1990; Klich and Pitt 1988; Pitt et al. 2000; Klich 2002; Samson and Varga 2009). However, their diversity was historically underestimated due to the high interspecific similarity of some species in this section. With the shift in species concepts toward phylogenetic species recognition and the increasing identification of cryptic species, a polyphasic approach has become essential for accurate taxonomy in this group. This integrative strategy enables more precise species delimitation and a better understanding of phylogenetic relationships (Varga et al. 2011; Samson et al. 2014; Frisvad et al. 2019; Norlia et al. 2019). In light of these advances, strains preserved in the URM collection must be sequenced to ensure alignment with current taxonomic standards. Currently, the section *Flavi* harbors 35 species distributed among eight series (Frisvad et al. 2019; Gilchrist et al. 2020; Visagie et al. 2024).

One of the main interests of the URM culture collection is to improve the quality and accuracy of its accessions. Accordingly, one of the main goals of the URM culture collection is to re-identify its strains using molecular data to improve taxonomic accuracy. Additionally, the collection has explored the biotechnological potential of its strains, particularly for producing enzymes with industrial and pharmaceutical applications. Recent studies have highlighted the use of L-asparaginase (e.g. Silva et al. 2022a, b; Pádua et al. 2024). Despite the recognized importance of *Aspergillus* section *Flavi*, comprehensive studies integrating taxonomic re-identification and biotechnological evaluation of preserved strains—particularly in Brazilian fungal collections—remain scarce.

This study aims to address this gap by applying a molecular phylogenetic approach to strains stored in the URM culture collection. Strains of *Aspergillus* section *Flavi* preserved from 1954 to 2021 were re-identified based on DNA sequence data (*BenA* and *CaM*). The primary aim of this work was to re-identify these strains and evaluate the kojic acid production potential of at least one representative strain from each identified species, using sugarcane molasses and corn steep liquor as substrates. Given that the chemical composition of sugarcane molasses can significantly influence fungal metabolism and product yield, its physicochemical characterization was performed to assess its suitability for kojic acid production.

## Materials and methods

### Strains

The *Aspergillus* section *Flavi* strains used in this study were obtained from the URM culture collection (WDCM 604), hosted at the Departamento de Micologia, Universidade Federal de Pernambuco, Recife, Brazil. Following reactivation attempts, most of the strains—originally preserved under mineral oil—were viable, with 198 showing growth. The strains used were isolated from different substrates/hosts. All strains were initially reactivated in 2 mL of glucose broth (Lacaz et al. 2002) and subsequently transferred to malt extract agar (MEA; HIMEDIA) before proceeding with the next stages of study. Table 1 summarizes the strain information obtained from the URM database.

**Table 1:** Strains from the URM culture collection and GenBank accession numbers of *Aspergillus* section *Flavi* analyzed in this study

URM Number	Strains name as registered	Current name	Deposit year	Host/substrate	*BenA*	*CaM*
36	*A. flavus*	*A. flavus*	1954	Unknown	PP965212	PP940367
135	*A. oryzae*	*A. tamarii*	1954	Unknown	PP940305	PP940493
221	*A. flavus*	*A. flavus*	1957	Unknown	PP965213	PP940368
421	*A. flavus*	*A. pseudocaelatus*	1957	*Bertholetia* sp.	PP940193	PP940344
433	*A. oryzae*	*A. tamarii*	1955	Air	PP940306	PP940494
648	*A. flavus*	*A. novoparasiticus*	1956	Otomycosis secretion	PP940304	PP940492
753	*A. parasiticus*	*A. parasiticus*	1957	Unknown^a^	PP940296	PP940484
951	*A. sojae*	*A. flavus*	1957	Unknown^b^	PP965214	PP940369
1035	*A. tamarii*	*A. minisclerotigenes*	1958	Unknown	PP940200	PP940351
1869	*A. flavus*	*A. flavus*	1965	Soil	PP965215	PP940370
1873	*A. oryzae*	*A. nomiae*	1963	Soil	PP940202	PP940353
1878	*A. oryzae*	*A. nomiae*	1963	Unknown	PP940203	PP940354
2161	*A. flavus*	*A. tamarii*	1966	*Amejuba* sp.	PP940307	PP940495
2226	*A. flavus*	*A. flavus*	1958	Bronchial lavage	PP965216	PP940371
2232	*A. flavus*	*A. tamarii*	1968	Bird	PP940308	PP940496
2467	*A. flavus*	*A. flavus*	1973	Unknown	PP965217	PP940372
2487	*A. flavus*	*A. flavus*	1978	Leaf litter	PP965218	PP940373
2578	*A. flavus*	*A. flavus*	1980	Soybean seeds	PP965219	PP940374
2579	*A. parasiticus*	*A. parasiticus*	1980	Unknown^a^	PP940297	PP940485
2581	*A. parasiticus*	*A. arachidicola*	1980	Atmospheric air	PV093537	PV037242
2582	*A. flavus*	*A. flavus*	1980	Atmospheric air^a^	PP965220	PP940375
2701	*A. flavus*	*A. flavus*	1981	Contaminant	PP965221	PP940376
2726	*A. flavus*	*A. flavus*	1982	Unknown	PP965222	PP940377
2814	*A. flavus*	*A. saccharicola*	1985	Sugarcane bagasse	PP940214	PP940365
2854	*A. parasiticus*	*A. nomiae*	1986	*Diatracea saccharalis*	PP940204	PP940355
2880	*A. parasiticus*	*A. flavus*	1986	Unknown	PP965223	PP940378
3209	*A. alliaceus*	*A. tamarii*	1990	Unknown^a^	PP940309	PP940497
3266	*A. tamarii*	*A. tamarii*	1991	Brazil nut	PP940310	PP940498
3416	*A. parasiticus*	*A. flavus*	-	Oat	PP965224	PP940379
3426	*A. flavus*	*A. flavus*	1994	Oat	PP965225	PP940380
3429	*A. parasiticus*	*A. flavus*	1994	Oat	PP965226	PP940381
3434	*A. parasiticus*	*A. flavus*	1994	Processed Oat	PP940295	PP940483
3442	*A. parasiticus*	*A. flavus*	1994	Processed Oat	PP965227	PP940382
3448	*A. parasiticus*	*A. flavus*	1994	Processed Oat	PP965228	PP940383
3454	*A. flavus*	*A. flavus*	1994	Bird excrement	PP965229	PP940384
3463	*A. parasiticus*	*A. flavus*	1994	Oat	PP965230	PP940385
3469	*A. flavus*	*A. flavus*	1994	Sawdust Pile	PP965231	PP940386
3488	*A. parasiticus*	*A. tamarii*	1994	Bird excrement	PP940311	PP940499
3499	*A. flavus*	*A. arachidicola*	1994	Rhizosphere of *Vernonia discolor*	PV093538	PV037243
3688	*A. flavus*	*A. flavus*	1996	Foliage	PP965232	PP940387
3739	*A. flavus*	*A. flavus*	1997	Water	PP965233	PP940388
4217	*A. tamarii*	*A. tamarii*	2000	Air	PP940312	PP940500
4242	*A. caelatus*	*A. caelatus*	2000	Soil^a^	PP940195	PP940346
4243	*A. nomiae*	*A. nomiae*	2000	Unknown^c^	PP940205	PP940356
4253	*A. flavus*	*A. flavus*	2000	Air	PP965234	PP940389
4365	*A. flavus*	*A. flavus*	2001	Soil	PP965235	PP940390
4509	*A. flavus*	*A. flavus*	2002	Soil	PP965236	PP940391
4522	*A. tamarii*	*A. tamarii*	2002	Pre-cooked cornmeal	PP940313	PP940501
4540	*A. flavus*	*A. flavus*	2002	Crushed corn (coarse cornmeal)	PP965237	PP940392
4541	*A. flavus*	*A. flavus*	2002	Corn flour	PP965238	PP940393
4634	*A. tamarii*	*A. tamarii*	2003	Soil	PP940314	PP940502
4687	*A. flavus*	*A. luteovirescens*	2003	Cassava gum	PV093533	PV037238
4709	*A. flavus*	*A. pseudocaelatus*	2003	Soil	PP940196	PP940347
4876	*A. tamarii*	*A. tamarii*	2004	Industrial residue	PP940315	PP940503
4928	*A. flavus*	*A. flavus*	2005	Soil	PP965239	PP940394
4933	*A. parasiticus*	*A. flavus*	2005	Soil	PP965240	PP940395
4992	*A. tamarii*	*A. tamarii*	2005	Chamomile tea	PP940316	PP940504
4999	*A. flavus*	*A. flavus*	2005	Tea	PP965241	PP940396
5051	*A. avenaceus*	*A. flavus*	2005	Soil	PP965242	PP940397
5167	*A. flavus*	*A. flavus*	2005	Human skin	PP965243	PP940398
5244	*A. tamarii*	*A. tamarii*	2006	Rhizosphere of *Mimosa* sp.	PP940317	PP940505
5265	*A. tamarii*	*A. tamarii*	2006	Rhizosphere of *Croton* sp.	PP940318	PP940506
5362	*A. tamarii*	*A. tamarii*	2006	Water	PP940319	PP940507
5364	*A. flavus*	*A. flavus*	2006	Unknown	PP965244	PP940399
5405	*A. flavus*	*A. flavus*	-	Shrimp shell	PP965245	PP940400
5408	*A. parasiticus*	*A. flavus*	2006	Shrimp shell	PP965246	PP940401
5427	*A. flavus*	*A. flavus*	2007	Water	PP965247	PP940402
5463	*A. oryzae*	*A. tamarii*	2007	Unknown	PP940320	PP940508
5491	*A. tamarii*	*A. flavus*	2007	Unknown	PP965248	PP940403
5493	*A. flavus*	*A. flavus*	2007	Peanut flour	PP940216	PP940404
5511	*A. parasiticus*	*A. flavus*	2007	Unknown	PP940217	PP940405
5533	*A. parasiticus*	*A. parasiticus*	2007	Unknown	PP940298	PP940486
5534	*A. oryzae*	*A. parasiticus*	2007	Unknown	PP940299	PP940487
5564	*A. tamarii*	*A. tamarii*	2007	Mangrove sediment	PP940321	PP940509
5575	*A. tamarii*	*A. tamarii*	2007	Water	PP940322	PP940510
5585	*A. parasiticus*	*A. flavus*	2007	Water	PP940218	PP940406
5600	*A. flavus*	*A. nomiae*	2007	Adult shrimp	PP940206	PP940357
5621	*A. flavus*	*A. flavus*	2007	Industrial castor pie	PP940219	PP940407
5638	*A. oryzae*	*A. flavus*	2007	Water from adult shrimp pond	PP940220	PP940408
5647	*A. flavus*	*A. flavus*	2007	Water from adult shrimp pond	PP940221	PP940409
5653	*A. tamarii*	*A. tamarii*	2007	Sunflower pie	PP940323	PP940511
5659	*A. flavus*	*A. flavus*	2007	Water from adult shrimp pond	PP940222	PP940410
5662	*A. flavus*	*A. flavus*	2007	Water from adult shrimp pond	PP940223	PP940411
5663	*A. flavus*	*A. flavus*	2007	Water from adult shrimp pond	PP940224	PP940412
5703	*A. flavus*	*A. flavus*	-	Seawater	PP940225	PP940413
5706	*A. parasiticus*	*A. flavus*	-	Seawater	PP940226	PP940414
5740	*A. flavus*	*A. flavus*	2008	Industrial castor pie	PP940227	PP940415
5778	*A. parasiticus*	*A. tamarii*	-	Laboratory castor pie	PP940324	PP940512
5787	*A. parasiticus*	*A. nomiae*	-	Industrial castor pie^c^	PP940207	PP940358
5791	*A. flavus*	*A. minisclerotigenes*	2008	Industrial castor pie	PP940201	PP940352
5793	*A. flavus*	*A. flavus*	2008	Industrial castor pie	PP940228	PP940416
5794	*A. flavus*	*A. flavus*	2008	Soil	PP940229	PP940417
5855	*A. flavus*	*A. pseudonomiae*	2007	Soil	PP940208	PP940359
5865	*A. parasiticus*	*A. nomiae*	2008	Industrial castor pie	PP940209	PP940360
5915	*A. parasiticus*	*A. flavus*	2009	Tracheal Secretion	PP940230	PP940418
5963	*A. parasiticus*	*A. caelatus*	-	Soil	PP940197	PP940348
5985	*A. flavus*	*A. luteovirescens*	2009	Human cadaver lung	PV093534	PV037239
5987	*A. flavus*	*A. luteovirescens*	2009	Human cadaver lung	PV093535	PV037240
6029	*A. flavus*	*A. luteovirescens*	-	Brain	PV093536	PV037241
6030	*A. tamarii*	*A. nomiae*	-	Lung	PP940210	PP940361
6065	*A. tamarii*	*A. tamarii*	2009	Mangrove sediment	PP940325	PP940513
6070	*A. parasiticus*	*A. flavus*	2009	Mangrove sediment	PP940231	PP940419
6300	*A. flavus*	*A. flavus*	-	Unknown	PP940232	PP940420
6301	*A. flavus*	*A. flavus*	-	Unknown	PP940233	PP940421
6302	*A. flavus*	*A. flavus*	-	Unknown	PP940234	PP940422
6303	*A. flavus*	*A. flavus*	-	Unknown	PP940235	PP940423
6305	*A. flavus*	*A. flavus*	-	Unknown	PP940236	PP940424
6306	*A. flavus*	*A. flavus*	-	Unknown	PP940237	PP940425
6309	*A. flavus*	*A. flavus*	-	Unknown	PP940238	PP940426
6310	*A. flavus*	*A. flavus*	-	Unknown	PP940239	PP940427
6313	*A. flavus*	*A. flavus*	-	Sputum	PP940240	PP940428
6314	*A. flavus*	*A. flavus*	-	Sputum	PP940241	PP940429
6315	*A. flavus*	*A. flavus*	-	Sputum	PP940242	PP940430
6322	*A. tamarii*	*A. tamarii*	2011	Otomycosis secretion	PP940326	PP940514
6323	*A. parasiticus*	*A. flavus*	2011	Otomycosis secretion	PP940243	PP940431
6324	*A. parasiticus*	*A. flavus*	2011	Otomycosis secretion	PP940244	PP940432
6332	*A. flavus*	*A. flavus*	2011	Otomycosis secretion	PP940245	PP940433
6333	*A. flavus*	*A. flavus*	2011	Otomycosis secretion	PP940246	PP940434
6434	*A. flavus*	*A. flavus*	2011	Artichoke solution - Herbal medicine	PP940247	PP940435
6435	*A. tamarii*	*A. tamarii*	2011	Horse Chestnut pill	PP940327	PP940515
6442	*A. flavus*	*A. flavus*	2011	Endophytic fungi from Cactaceae	PP940248	PP940436
6480	*A. flavus*	*A. flavus*	2011	Artichoke solution - Herbal medicine	PP940249	PP940437
6481	*A. flavus*	*A. flavus*	2011	Capsule of horse chestnut - Herbal medicine	PP940250	PP940438
6482	*A. flavus*	*A. flavus*	2011	Artichoke solution - Herbal medicine	PP940251	PP940439
6565	*A. flavus*	*A. flavus*	2012	Sputum	PP940252	PP940440
6574	*A. flavus*	*A. flavus*	2012	Sputum	PP940253	PP940441
6594	*A. tamarii*	*A. tamarii*	2012	Soil	PP940328	PP940516
6599	*A. tamarii*	*A. tamarii*	2012	Soil	PP940329	PP940517
6602	*A. flavus*	*A. flavus*	2012	Soil	PP940254	PP940442
6621	*A. flavus*	*A. flavus*	2012	Soil	PP940255	PP940443
6643	*A. flavus*	*A. flavus*	2012	Soil	PP940256	PP940444
6706	*A. avenaceus*	*A. flavus*	2012	Soil	PP940257	PP940445
6718	*A. tamarii*	*A. tamarii*	2012	Dog	PP940330	PP940518
6720	*A. tamarii*	*A. tamarii*	2012	Dog	PP940331	PP940519
6745	*A. tamarii*	*A. tamarii*	2012	Otomycosis secretion	PP940332	PP940520
6746	*A. tamarii*	*A. tamarii*	2012	Otomycosis secretion	PP940333	PP940521
6751	*A. tamarii*	*A. tamarii*	2012	Sediment contaminated	PP940334	PP940522
6825	*A. parasiticus*	*A. flavus*	2012	Soil	PP940258	PP940446
6867	*A. parasiticus*	*A. nomiae*	2012	Endophytic fungi	PP940211	PP940362
6868	*A. parasiticus*	*A. nomiae*	2012	Endophytic fungi	PP940212	PP940363
6886	*A. flavus*	*A. flavus*	2012	Endophytic fungi	PP940259	PP940447
6887	*A. flavus*	*A. flavus*	2012	Endophytic fungi	PP940260	PP940448
6958	*A. tamarii*	*A. tamarii*	2013	Dog	PP940335	PP940523
6959	*A. tamarii*	*A. tamarii*	2013	Dog	PP940336	PP940524
7001	*A. tamarii*	*A. tamarii*	2013	Soil	PP940337	PP940525
7002	*A. flavus*	*A. flavus*	2013	Soil	PP940261	PP940449
7009	*A. caelatus*	*A. pseudocaelatus*	2013	Soil	PP940198	PP940349
7022	*A. caelatus*	*A. pseudocaelatus*	2013	Soil	PP940199	PP940350
7028	*A. flavus*	*A. flavus*	2013	Soil	PP940262	PP940450
7101	*A. tamarii*	*A. flavus*	2013	Sediment and ocean waters	PP940263	PP940451
7113	*A. flavus*	*A. flavus*	2013	*Syzygium cumini* leaves	PP940264	PP940452
7115	*A. tamarii*	*A. flavus*	2013	*Syzygium cumini* leaves	PP940265	PP940453
7172	*A. flavus*	*A. pseudocaelatus*	2014	*Vitis labrusca* rhizoplane	PP940194	PP940345
7194	*A. tamarii*	*A. tamarii*	2014	Rhizosphere of *Vitis labrusca*	PP940338	PP940526
7257	*A. flavus*	*A. flavus*	2015	Surface of Buriti nut	PP940266	PP940454
7258	*A. flavus*	*A. flavus*	2015	Buriti Flower	PP940267	PP940455
7262	*A. flavus*	*A. flavus*	2015	Soil	PP940268	PP940456
7268	*A. parasiticus*	*A. flavus*	2015	Leaves	PP940269	PP940457
7271	*A. tamarii*	*A. tamarii*	2015	Unknown	PP940339	PP940527
7272	*A. flavus*	*A. flavus*	2015	Oil and grease	PP940270	PP940458
7273	*A. flavus*	*A. flavus*	2015	Oil and grease	PP940271	PP940459
7274	*A. parasiticus*	*A. flavus*	2015	Oil and grease	PP940272	PP940460
7276	*A. flavus*	*A. flavus*	2015	Oil and grease	PP940273	PP940461
7277	*A. flavus*	*A. flavus*	2015	Oil and grease	PP940274	PP940462
7286	A. parasiticus	A. flavus	2015	Soil	PP940275	PP940463
7288	*A. parasiticus*	*A. saccharicola*	2015	Soil	PP940215	PP940366
7291	*A. flavus*	*A. flavus*	2015	Not specified	PP940276	PP940464
7295	*A. flavus*	*A. flavus*	2015	Spoiled bread	PP940277	PP940465
7308	*A. parasiticus*	*A. parasiticus*	2015	Soil with decomposing material	PP940300	PP940488
7324	*A. flavus*	*A. flavus*	2016	*Brachiaria brizanta* cv. *Marandu*	PP940278	PP940466
7325	*A. flavus*	*A. flavus*	2016	*Brachiaria brizanta* cv. *Marandu*	PP940279	PP940467
7326	*A. flavus*	*A. flavus*	2016	*Brachiaria brizanta* cv. *Marandu*	PP940280	PP940468
7505	*A. tamarii*	*A. tamarii*	2016	*Opuntia ficus-indica*	PP940340	PP940528
7841	*A. flavus*	*A. flavus*	2018	Mineral water	PP940281	PP940469
7863	*A. flavus*	*A. flavus*	2018	Unknown	PP940282	PP940470
7937	*A. pseudonomiae*	*A. pseudonomiae*	2018	Otomycosis secretion	PP940213	PP940364
7938	*A. flavus*	*A. flavus*	2018	Otomycosis secretion	PP940283	PP940471
7939	*A. flavus*	*A. flavus*	2018	Otomycosis secretion	PP940284	PP940472
7940	*A. flavus*	*A. flavus*	2018	Otomycosis secretion	PP940285	PP940473
7942	A. tamarii	A. tamarii	2018	Otomycosis secretion	PP940341	PP940529
7943	*A. tamarii*	*A. tamarii*	2018	Otomycosis secretion	PP940342	PP940530
7945	*A. flavus*	*A. flavus*	2018	Otomycosis secretion	PP940286	PP940474
7949	*A. parasiticus*	*A. flavus*	2018	Mineral water	PP940287	PP940475
7960	*A. parasiticus*	*A. parasiticus*	2018	Otomycosis secretion	PP940301	PP940489
7961	*A. flavus*	*A. flavus*	2018	Otomycosis secretion	PP940288	PP940476
7962	*A. flavus*	*A. flavus*	2018	Otomycosis secretion	PP940289	PP940477
7967	*A. parasiticus*	*A. parasiticus*	2018	Cocoa beans	PP940302	PP940490
8184	*A. parasiticus*	*A. tamarii*	2019	Soil	PP940343	PP940531
8202	*A. flavus*	*A. flavus*	2019	Tile	PP940290	PP940478
8204	*A. tamarii*	*A. parasiticus*	2019	Tile	PP940303	PP940491
8274	*A. flavus*	*A. flavus*	2019	Tile	PP940291	PP940479
8345	*A. oryzae*	*A. flavus*	2019	Soil	PP940292	PP940480
8536	*A. flavus*	*A. flavus*	2021	Strawberry	PP940293	PP940481
8537	*A. flavus*	*A. flavus*	2021	Strawberry	PP940294	PP940482

Most of the analyzed strains originate from Brazil, except: a United States; b Japan; c Unknown. Additional information (http://www3.ufpe.br/micoteca/nova/fazerBusca.php)

### DNA extraction, polymerase chain reaction, and sequencing

The Wizard Genomic DNA Purification Kit (Promega) was used to isolate genomic DNA from 7-day-old colonies grown on MEA. Initially, the calmodulin (*CaM*) gene primers were amplified, followed by amplification of the β-tubulin (*BenA*) gene. The primer pairs used were CMD5/CMD6 and Bt2a/Bt2b, as described by Houbraken et al. (2012) and Samson et al. (2014). PCR products were purified using Exonuclease/Alkaline Phosphatase mix (Cellco Biotec.), according to the manufacturer’s instructions. Sequencing was performed using the same primers and the BigDye^®^ Terminator v.3.1 Cycle Sequencing Kit (Applied Biosystems Life Technologies, Carlsbad, CA, USA) on the Multi-User Sequencing and Gene Expression Platform at the Center for Biological Sciences, UFPE.

### Phylogenetic analyses

Electropherograms were analyzed using MEGA v.7 (Kumar et al. 2016), which was also used to generate consensus sequences. The generated nucleotide sequences were edited in MEGA v.7 (Kumar et al. 2016) and compared with GenBank data using the BLASTn tool. Alignments containing newly generated sequences together with reference sequences—preferably from ex-type strains—retrieved from previously published studies (Frisvad et al. 2019; Gilchrist et al. 2020; Houbraken et al. 2020; Visagie et al. 2024) were aligned using MAFFT v.7 (Katoh and Standley 2013). Alignments were reviewed and, when necessary, adjusted using MEGA v.7 (Kumar et al. 2016). Initially, phylogenetic relationships among accepted species of section *Flavi* and all viable URM strains were determined by individual analyses of *CaM* and *BenA* sequence alignments, followed by a concatenated analysis of both markers. Individual alignments were concatenated using Mesquite v.3.04 (Maddison and Maddison 2015).

The most suitable substitution model for each dataset was determined using jModelTest v.2.1.7 (Posada 2008). Maximum likelihood (ML) phylogenetic analysis was conducted using RAxML-HPC v.8.2.8 BlackBox, with 1,000 rapid bootstrap replicates. Bayesian inference (BI) analysis was conducted using MrBayes v.3.2.7a (Ronquist et al. 2012) on XSEDE through the CIPRES Science Gateway portal (Miller et al. 2010), with 5 × 10^6^ generations, a burn-in value of 25%, and chains sampled every 5,000 generations. Values equal to or greater than 0.95 BI posterior probability (BPP) and 70% ML bootstrap support (ML-BS) were shown next to the nodes. Phylogenetic trees were visualized in FigTree v.1.4.4 (Rambaut 2010) and edited in Adobe Illustrator CS5.1. Branches with full support (PP = 1.00 and ML-BS = 100%) were thickened.

### Kojic acid screening

Strains from each species, isolated from different substrates, were selected for screening of kojic acid production. The strains were selected based on their morphological characteristics (good sporulation and growth) and phylogenetic position. Kojic acid production was screened using the rapid detection method described by Chib et al. (2019). Mycelial fragments of the grown strains were suspended in 2 mL of a solution containing 0.1% Tween 80. Then, 5 µL of this solution was inoculated at the center of Petri dishes containing PDA medium (KASVI). The Petri dishes were incubated in the dark at 28 ± 2 °C for 5 days.

Based on the results of the qualitative assay on PDA, strains that exhibited good growth, sporulation, and kojic acid production were selected for further testing using an alternative, low-cost solid medium composed of sugar cane molasses, corn steep liquor, and agar (SMC) was tested. The SMC agar medium was prepared using sugarcane molasses (40 g/L) as the carbon source and corn steep liquor (5 g/L) as the nitrogen source, with 20 g/L agar. All components were dissolved in 1 L of distilled water and adjusted to pH 3. This alternative medium was tested to assess the production potential of the selected strains under cost-effective conditions before progressing to liquid fermentation assays.

### Fermentation using agro-industrial waste

For the quantitative analysis, twelve strains were cultivated in 250 ml Erlenmeyer flasks containing 100 ml of SMC broth. The medium was prepared with a final pH adjusted to 3.0 and sterilized. To prepare the inoculum, fungal hyphae and spores were scraped and suspended in sterile water containing 0.01% (v/v) Tween 80. The suspension was stirred to ensure homogeneity and adjusted to a final concentration of 3 × 10^5^ spores/ml. Cultures were incubated at 30 ± 2 °C under agitation at 150 rpm for eight days in the dark. All experiments were performed independently in duplicate. During the incubation period, samples were collected at four time points (days 0, 2, 4, 6, and 8). For each time point, two independent replicates (*n* = 2) were analyzed. At each sampling point, 500 µL aliquots of the supernatant were collected, centrifuged to remove residual biomass, and transferred to a clean microtube. Samples were stored at − 20 °C until analysis of kojic acid and total carbohydrate concentrations.

### Origin of agro-industrial waste

Sugarcane molasses obtained from the Usina Alcoolquímica, an alco-chemical production facility that is part of the JB Group (Vitória de Santo Antão, Pernambuco, Brazil), was stored at 4 °C until. Physicochemical analyses and experimental use in kojic acid production. The experiments were conducted at the Laboratory of Biotechnological Processes (Laboratório de Processos e Produtos Biotecnológicos) and Products and the Laboratory of Microbial Metabolism (Laboratório de Metabolismo Microbiano), both part of the Institute of Biological Sciences at the University of Pernambuco (Instituto de Ciências Biológicas da Universidade de Pernambuco, Recife, Brazil). Corn steep liquor was treated before analysis. The pH was adjusted to 8.0 using 4 M sodium hydroxide (NaOH), as described by Liggett and Koffler (1948). After pH adjustment, the liquor was incubated in a water bath at 80 °C for 1 h, followed by centrifugation at 1700 × g for 15 min. The resulting supernatant was collected and stored at − 20 °C until further analyses (Santana et al. 2021).

### Physicochemical characterization of sugarcane molasses

To better understand the nutritional composition and its potential for use as a substrate in the production of kojic acid, the physicochemical properties of sugarcane molasses were characterized. The physicochemical parameters of sugarcane molasses were determined according to the methods described by Adolfo Lutz Institute (Instituto Adolfo Lutz 2008). The following analyses were performed on sugarcane molasses: humidity (drying in an oven at 105 °C for 3 h), ash (incineration at 550 °C for 4 h), pH (measured after dilution using a TEC-7 Potentiometer), and free acidity (titration with 0.05 N NaOH). The brix (ºBrix) was measured using a portable refractometer, and the density was determined with a 5 mL glass pycnometer previously calibrated with distilled water.

The determination of carbohydrate concentrations (glucose, fructose and sucrose) was based on high-performance liquid chromatography (HPLC) in an Agilent system (Agilent Technologies 1200 Series) equipped with a quaternary pump coupled to a degasser (model G1322A), an injector detector (model G1329A) and a refractive index (RID) detector (model G1362A). Samples were diluted with deionized water acidified with 5 mM sulfuric acid (H_2_SO_4_) and filtered through a 0.22 μm membrane. An ion exchange column (Aminex^®^ HPX-87 H, Bio-Rad, USA; 300 × 7.8 mm, 9 μm particle size), was used. The mobile phase was 5 mM H_2_SO_4_ at a flow rate of 0.6 mL.min^− 1^. Column temperature was maintained at 35 °C, and the sample injection volume was 20 µL. Metabolites were identified by their retention times and quantified using calibration curves generated with external standards. Results are the average of at least two technical replicates.

### Determination of dry biomass

At the end of the incubation period, the fungal mycelia were recovered by filtration using quantitative filter paper (2 µ, Química Moderna). The biomass was washed with distilled water and then dried in an oven at 90 °C until a constant weight was achieved, as verified using an analytical balance. The culture supernatants were collected and stored for the quantitative analysis of kojic acid and the evaluation of residual carbohydrate content.

### Determination of kojic acid, total carbohydrates, and residual carbohydrates

Kojic acid concentrations were determined by HPLC using an Agilent Technologies 1200 Series system equipped with a binary pump, degasser (model G1322A), an automatic injector (model G1329A), and a diode-array detector (DAD, model G1315C) set at 280 nm. Samples were diluted in ultrapure water and acetonitrile (80:20 v/v). Separation was carried out on a C18 300 A column (Jupiter, 250 × 4.60 mm, 5 μm) using an isocratic mobile phase of ultrapure water and acetonitrile (80:20, v/v) at a flow rate of 0.7 mL.min^− 1^. The column temperature was maintained at 25 °C, and the injection volume was 50 µL (Eradati et al. 2021). Kojic acid was identified by its retention time and quantified using an external calibration curve. Results were expressed as the average of two biological replicates. Yield (Y = ΔP/ΔS) was calculated as grams of kojic acid per gram of dry biomass (g/g), providing insight into production efficiency.

Total carbohydrate content in the samples was determined using an Amined HPX-87 H column (BioRad, USA) maintained at 35 °C, with a 5 mM sulfuric acid solution as the mobile phase at a flow rate of 0.6 L min^− 1^. For analyses, 2.5 g of each sugarcane molasses sample was diluted in 100 mL of distilled water. To determine residual carbohydrate concentrations in fermentation supernatants, aliquots were diluted in deionized water and analyzed under the same chromatographic conditions. All carbohydrate analyses were performed in triplicate.

### Statistical analysis

Kojic acid concentrations were obtained from biological duplicates and analyzed by one-way analysis of variance (ANOVA), followed by Tukey’s test to compare means on the day of highest kojic acid production for most of the fungal strains. All statistical analyses were performed using Minitab 17 at a 5% significance level (*p* ≤ 0.05).

## Results

### Phylogeny of *Aspergillus* section *Flavi* at micoteca URM in Brazil

We analyzed 198 strains stored in the URM culture collection, which were originally identified as belonging to the *Aspergillus* section *Flavi*. Following taxonomic re-evaluation based on molecular data, several strains were reassigned to different species or sections. Among the 105 strains initially identified as *A. flavus*, 88 retained this classification, while the remaining 17 were reassigned to *A. luteovirescens* (4), *A. pseudocaelatus* (3), *A. tamarii* (2), and one each to *A. minisclerotigenes*, *A. nomiae*, *A. novoparasiticus*, *A. pseudonomiae*, *A. saccharicola*, and *A. arachidicola*. Additionally, two strains were reclassified into section *Fumigati*. Of the 38 strains originally identified as *A. parasiticus*, only six retained that identity; the others were reassigned to *A. flavus* (21), *A. nomiae* (5), *A. tamarii* (3), and one each to *A. pseudocaelatus*, *A. saccharicola*, and *A. arachidicola*. Among the 37 *A. tamarii* strains, 30 remained unchanged, while the others were reclassified as *A. flavus* (3), *A. minisclerotigenes* (1), *A. nomiae* (1), *A. parasiticus* (1), and one strain was reassigned to section *Terrei* (Table 1).

Additional reclassification included one *A. alliaceus* strain reassigned to *A. tamarii* and another to section *Cremei*. The two strains previously identified as *A. avenaceus* were reidentified as *A. flavus*. Of the three strains originally classified as *A. caelatus*, one remained this classification, while the other two were reclassified as *A. pseudocaelatus*. The only strains previously identified as *A. nomiae* remained unchanged. Among the eight *A. oryzae* strains, three were reclassified as *A. tamarii*, two as *A. nomiae*, two as *A. flavus*, and one as *A. parasiticus*. The single strains of *A. pseudonomiae* and *A. sojae* were confirmed as *A. pseudonomiae* and reclassified as *A. flavus*, respectively.

Based on sequences of the *BenA* fragment and morphological features, four strains (URM 1875, URM 2580, URM 3072 and URM 3883) were excluded from the phylogenetic alignment, as they did not belong to section *Flavi*. Taxonomic reassessment indicated that URM 1875 belongs to section *Terrei*, URM 2580 and URM 3072 to section *Fumigati*, and URM 3883 to section *Cremei* (identified as *Aspergillus capibaribensis*, see Felipe et al. 2024). These strains were not included in the phylogenetic trees.

**Fig. 1:**
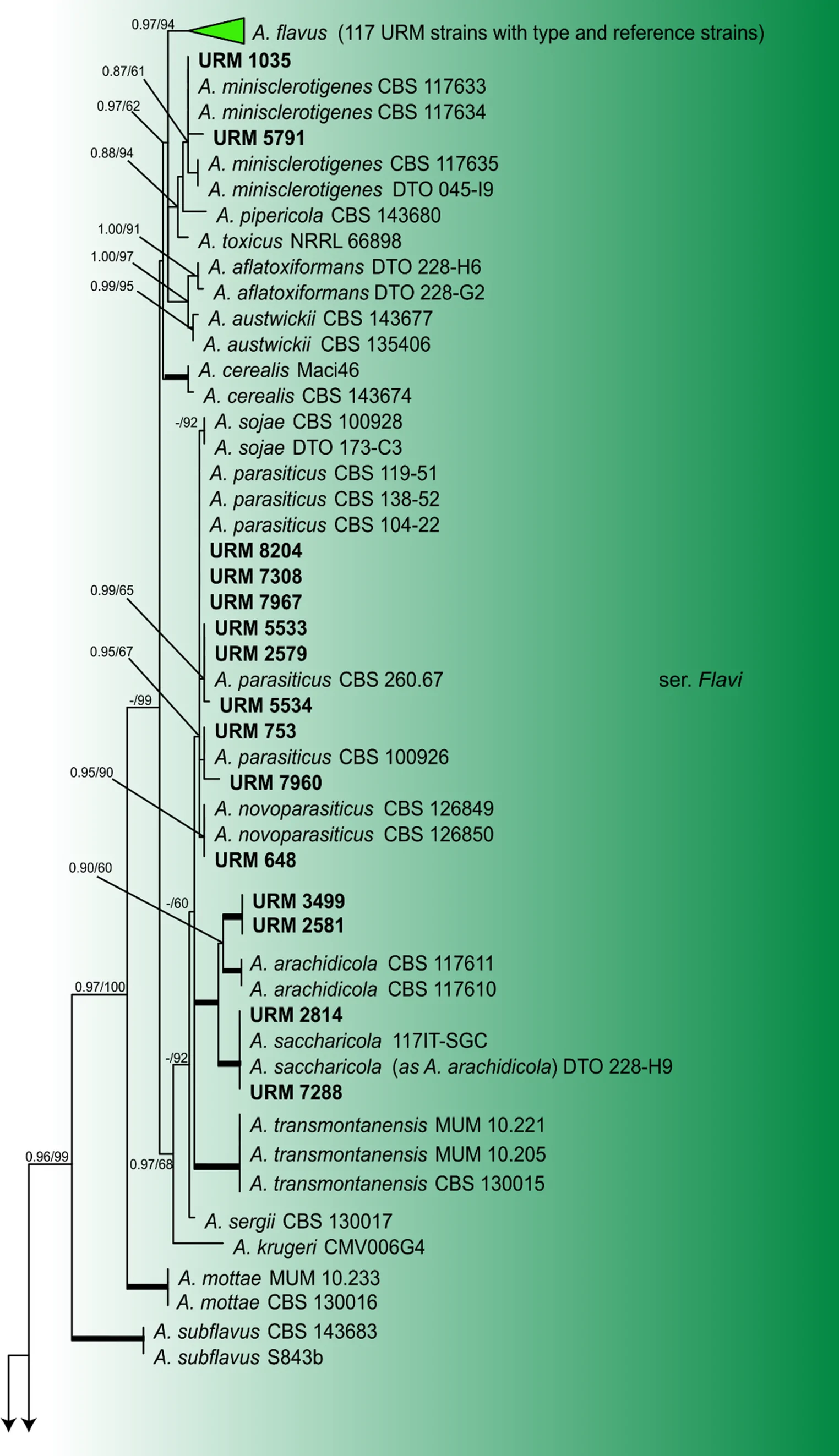

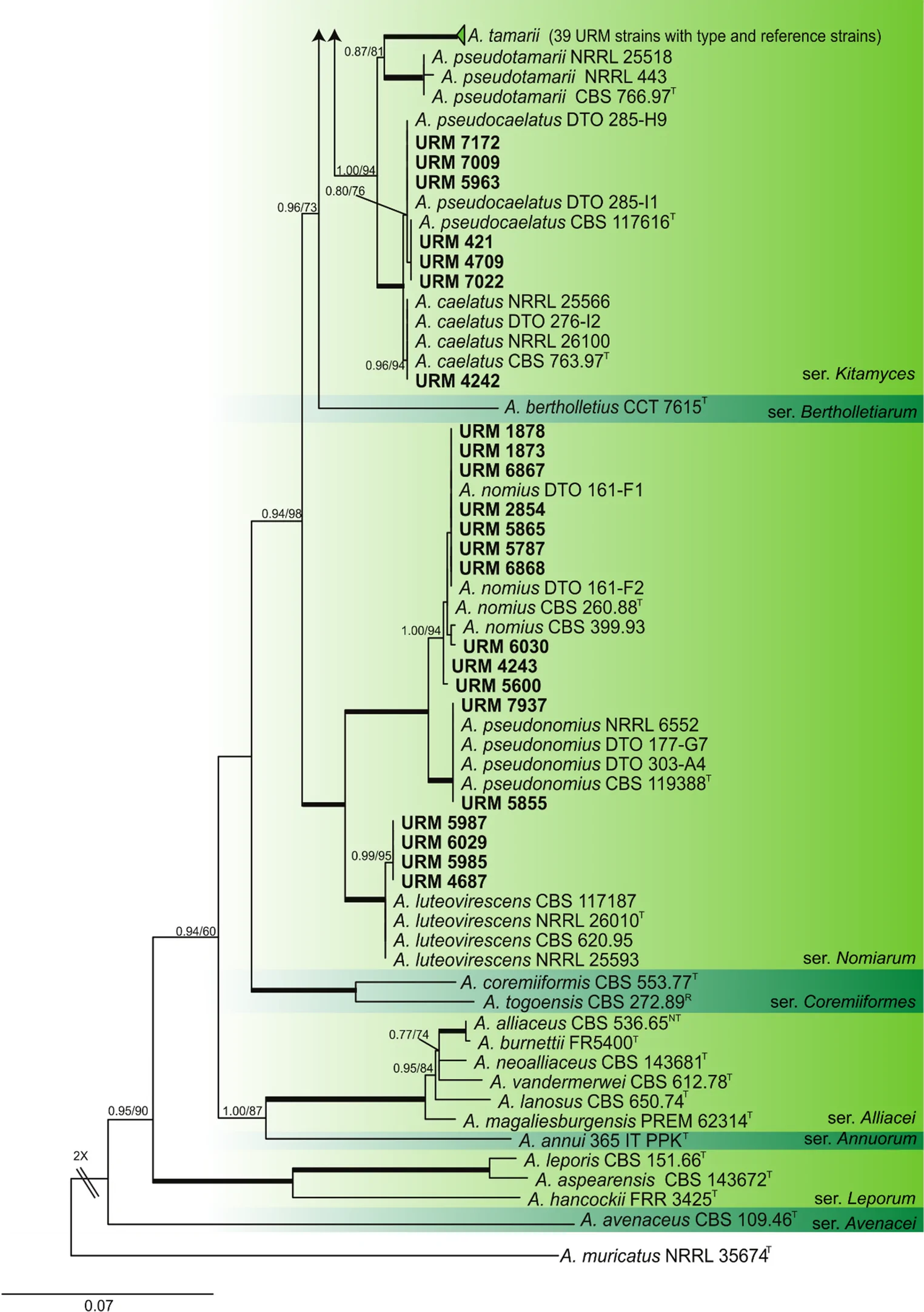
A phylogenetic tree based on Bayesian analysis was constructed using sequences of *CaM*-*BenA* of species included in *Aspergillus* section *Flavi*. Only bs values ≥ 60% and pp ≥ 0.95 are shown at branches. The branches that presented full statistical support (bs = 100% and pp = 1) are thickened. The strains used in this study are shown in bold. The tree was rooted to *Aspergillus muricatus* NRRL 35674

Phylogenetic relationships among URM strains and reference species were reconstructed using concatenated sequences of the *BenA* and *CaM* genes. The final dataset comprised 278 sequences: 194 from URM strains and 84 from publicly available (ex-)type strains, including *Aspergillus muricatus* NRRL35674 as the outgroup. The concatenated alignment contained 934 characters (*BenA* = 444, *CaM* = 490) (Fig. 1). ML analyses were performed using the GTRGAMA + I model, while BI employed TPM3 + I + G for *BenA* and JC + I+G for *CaM*. Individual analyses of the *BenA* and *CaM* gene regions are shown in Supplementary Figs. 1 and 2.

The resulting phylogeny revealed distinct clades: 114 strains grouped with the *A. flavus* strain ex-type, 39 with *A. tamarii*, 10 with *A. nomiae*, eight with *A. parasiticus*, six with *A. pseudocaelatus*, two with *A. saccharicola* and *A. caelatus*, four with *A. luteovirescens*, two with *A. arachidicola*, two with *A. pseudonomiae*, *A. minisclerotigenes*, and one with *A. novoparasiticus* (Table 1; Fig. 1).

### Qualitative screening of kojic acid-producing strains

To evaluate the influence of agro-industrial substrates on kojic acid production, a selection medium composed of sugarcane molasses, corn steep liquor, and agar (SMCA) was tested. Among the 99 *Aspergillus* section *Flavi* strains tested for their potential to produce kojic acid on solid medium, 46.46% (46 strains) grew and produced the acid on PDA (Supplementary Table 1). The chemical composition of the sugarcane molasses is presented in Supplementary Table 2. However, *Aspergillus tamarii* URM 3266 and *A. pseudonomiae* URM 7937, although displaying good growth and sporulation, did not produce kojic acid under the SMCA conditions. Although these strains did not produce kojic acid on SMCA, they produced it in liquid medium. Supplementary Fig. 3 shows representative *Aspergillus* strains capable of producing kojic acid on a solid medium.

### Kojic acid production in liquid medium

To quantify kojic acid production, twelve strains—each representing a distinct species within *Aspergillus* section *Flavi*—were tested: *Aspergillus flavus* URM 3739, *A. saccharicola* URM 7288, *A. caelatus* URM 4242, *A. luteovirescens* URM 4687, *A. nomiae* URM 6030, *A. parasiticus* URM 5534, *A. tamarii* 3266, *A. novoparasiticus* URM 648, *A. minisclerotigenes* URM 1035, *A. pseudocaelatus* URM 7009 and *A. pseudonomiae* URM 7937.

All strains grew and produced kojic acid at varying concentrations over the incubation period, except *A. arachidicola* URM 3499, which did not produce detectable levels under the tested conditions. The phrase “in varying concentrations” refers to the differences in kojic acid levels measured across strains and time points. Production was monitored over eight days, and the results represent the average of two independent replicates.

Among the strains analyzed, five showed concentrations of kojic acid (between 8 and 10.65 g/L), while another six obtained lower amounts (< 5 g/L). Generally, the highest kojic acid production was achieved after six days of incubation. Statistical analysis (Tukey’s test, *p* ≤ 0.05) showed that the highest kojic acid production was observed in *Aspergillus caelatus* URM 4242 (10.65 g/L), followed by *A. flavus* URM 3739 (10.44 g/L), and *A. nomiae* URM 6030 (9.74 g/L; Table 2). After four days of incubation, the highest kojic acid production was observed in *A. parasiticus* URM 5534, *A. flavus* URM 3739, and *A. luteovirescens* URM 4687, with production of 8.70 ± 0.0, 7.95 ± 0.7, and 7.44 ± 0.0 g/L, respectively.

**Table 2:** Production of kojic acid, total carbohydrates and biomass by strains of *Aspergillus* section *Flavi* deposited at URM culture collection (Recife, Brazil)

Strain	Fermentation time (days)	Kojic acid (g/L)	Total carbohydrates (g/L)	Biomass (g/L)
*A. novoparasiticus* URM 648	0	0.000 ± 0.0	19.76 ± 0.1	14.94 ± 0.0
	2	n.d.	17.98 ± 0.1	
	4	n.d.	12.12 ± 0.1	
	6	0.748 ± 0.0	8.554 ± 0.6	
	8	3.053 ± 0.1	3.022 ± 0.2	
*A. minisclerotigenes* URM 1035	0	0.000 ± 0.0	23.42 ± 0.1	14.86 ± 0.0
	2	n.d.	20.02 ± 0.2	
	4	n.d.	11.68 ± 0.2	
	6	n.d.	6.869 ± 0.2	
	8	0.668 ± 0.2	0.588 ± 0.1	
*A. tamarii* URM 3266	0	0.000 ± 0.0	19.76 ± 0.1	14.02 ± 0.0
	2	n.d.	15.37 ± 0.2	
	4	0.823 ± 0.2	8.566 ± 0.1	
	6	2.956 ± 0.7	3.871 ± 0.1	
	8	3.234 ± 0.2	0.000 ± 0.0	
*A. flavus* URM 3739	0	0.000 ± 0.0	19.76 ± 0.1	13.16 ± 0.0
	2	1.350 ± 0.4	14.53 ± 0.3	
	4	7.957 ± 0.7	6.446 ± 3.7	
	6	10.44 ± 0.3	0.000 ± 0.0	
	8	8.047 ± 0.5	0.000 ± 0.0	
*A. caelatus* URM 4242	0	0.000 ± 0.0	19.76 ± 0.1	13.98 ± 0.0
	2	0.131 ± 0.0	17.97 ± 0.0	
	4	6.110 ± 0.5	6.983 ± 0.7	
	6	10.65 ± 0.4	0.000 ± 0.0	
	8	8.840 ± 0.2	0.000 ± 0.0	
*A. luteovirescens* URM 4687	0	0.000 ± 0.0	19.76 ± 0.1	14.2 ± 0.0
	2	0.353 ± 0.0	15.60 ± 0.1	
	4	7.442 ± 0.0	0.773 ± 0.0	
	6	8.537 ± 0.0	0.000 ± 0.0	
	8	7.302 ± 0.3	0.000 ± 0.0	
*A. parasiticus* URM 5534	0	0.000 ± 0.0	19.76 ± 0.1	13.6 ± 0.0
	2	2.748 ± 0.2	12.96 ± 0.8	
	4	8.708 ± 0.0	0.679 ± 0.0	
	6	6.852 ± 0.2	0.000 ± 0.0	
	8	3.287 ± 0.3	0.000 ± 0.0	
*A. nomiae* URM 6030	0	0.000 ± 0.0	19.76 ± 0.1	14.12 ± 0.0
	2	2.748 ± 0.2	12.96 ± 0.8	
	4	8.708 ± 0.0	0.679 ± 0.0	
	6	6.852 ± 0.2	0.000 ± 0.0	
	8	3.287 ± 0.3	0.000 ± 0.0	
*A. pseudocaelatus* URM 7009	0	0.000 ± 0.0	23.42 ± 0.1	15.14 ± 0.0
	2	n.d.	16.62 ± 0.2	
	4	2.362 ± 0.0	8.435 ± 0.0	
	6	4.668 ± 0.1	6.205 ± 0.0	
	8	3.545 ± 0.0	0.000 ± 0.0	
*A. saccharicola* URM 7288	0	0.000 ± 0.0	19.76 ± 0.1	14.54 ± 0.0
	2	0.533 ± 0.0	15.36 ± 0.2	
	4	1.281 ± 0.0	8.535 ± 0.1	
	6	2.670 ± 0.1	4.649 ± 1.2	
	8	2.926 ± 0.4	0.000 ± 0.0	
*A. pseudonomiae* URM 7937	0	0.000 ± 0.0	23.42 ± 0.1	15.4 ± 0.0
	2	n.d.	19.54 ± 0.6	
	4	n.d.	12.73 ± 0.0	
	6	n.d.	2.736 ± 0.1	
	8	2.428 ± 0.0	0.000 ± 0.0	

n.d.: not detected; mean values ± standard deviation from two biological replicates; no significant differences were observed between replicates (*p* ≤ 0.05)

**Fig. 2:**
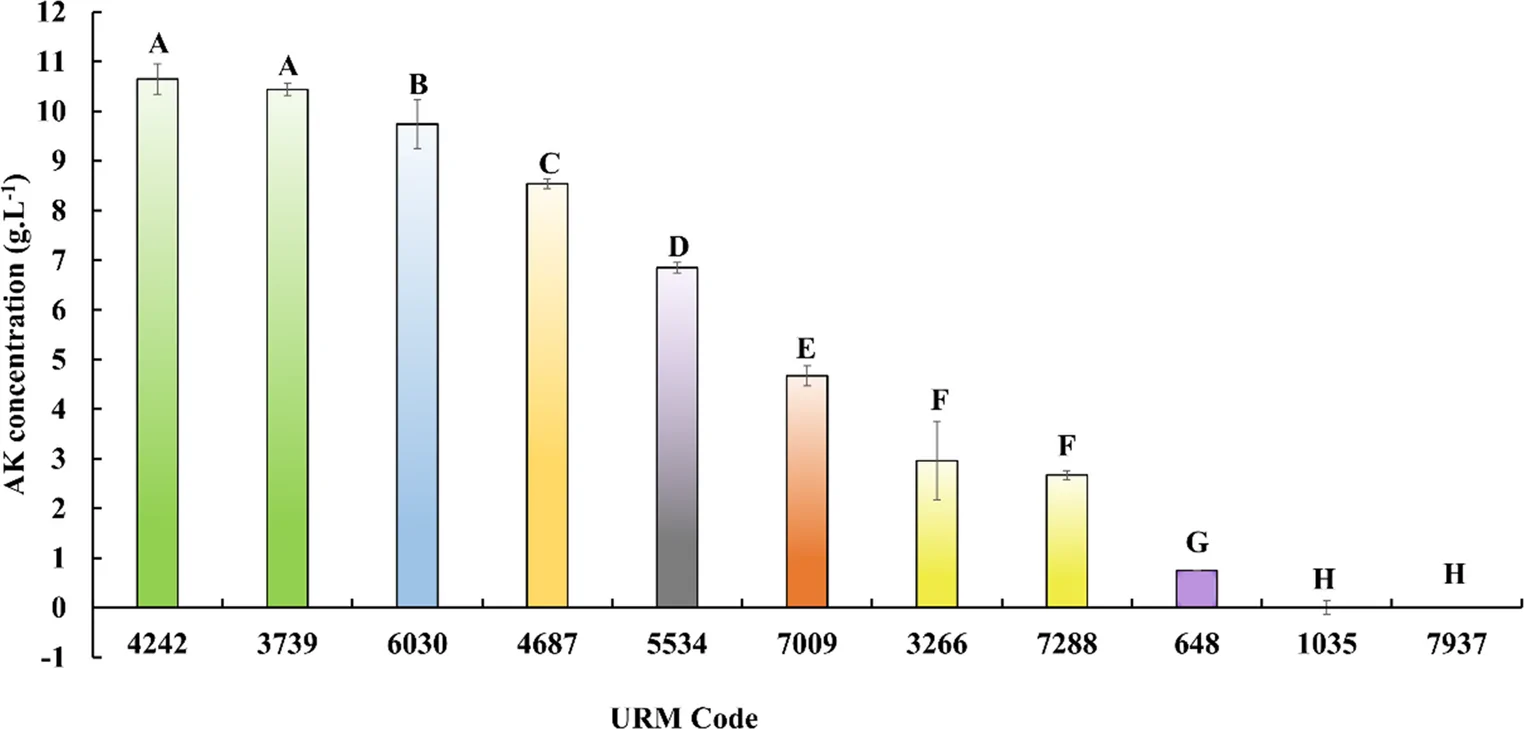
Production of kojic acid by different strains of *Aspergillus* section *Flavi* deposited at the URM culture collection (Recife, Brazil), using sugarcane molasses and corn steep liquor as substrate. Different letters (**A**, **B**, **C**, **D**, **E**, **F**, and **H**) indicate significant difference among the URM strains after six days of incubation (*p* ≤ 0.05). URM 4242 = *A. caelatus*; URM 3739 = *A. flavus*; URM 6030 = *A. nomiae*; URM 4687 = *A. luteovirescens;* URM 5534 = *A. parasiticus*; URM 7009 = *A. pseudocaelatus*; URM 3266 = *A. tamarii*; URM 7288 = *A. saccharicola*; URM 648 = *A. novoparasiticus*; URM 1035 = *A. minisclerotigenes*; and URM 7937 = *A. pseudonomiae*

**Fig. 3:**
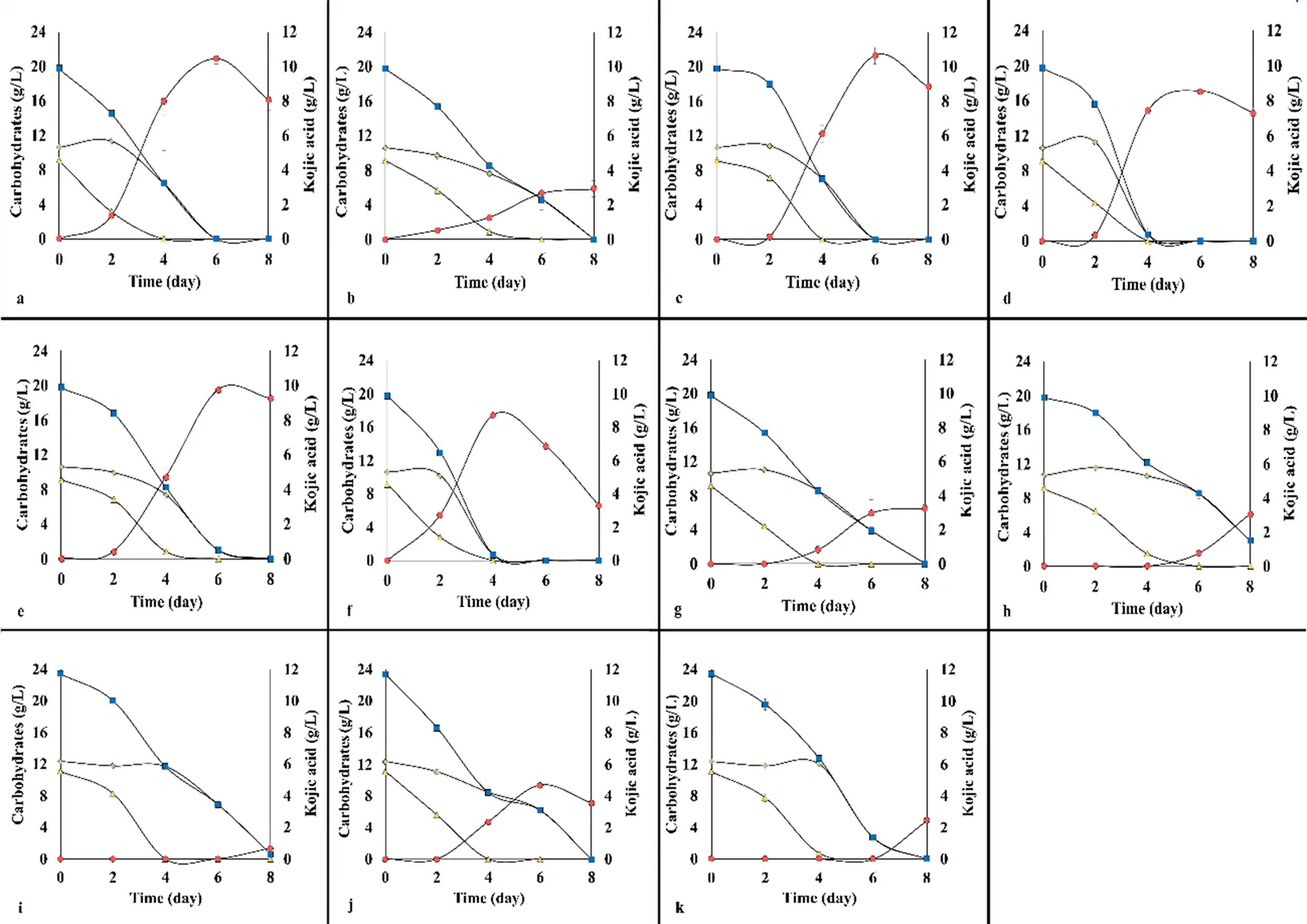
Relationship between total carbohydrate content in the medium and kojic acid production by each URM strain of *Aspergillus* section *Flavi*. **a** *A. flavus* URM 3739. **b** *A. saccharicola* URM 7288. **c** *A. caelatus* URM 4242. **d** *A. luteovirescens* URM 4687. **e** *A. nomiae* URM 6030. **f** *A. parasiticus* URM 5534. **g** *A. tamarii* URM 3266. **h** *A. novoparasiticus* URM 648. **i** *A. minisclerotigenes* URM 1035. **j** *A. pseudocaelatus* URM 7009. **k** *A. pseudonomiae* URM 7937

Notably, *A. parasiticus* URM 5534 reached 8.70 ± 0.0 g/L within just two days and peaked on day four, indicating rapid biosynthesis. In contrast, most other strains peaked on day six. By day eight, kojic acid concentrations declined slightly, likely due to compound degradation or nutrient depletion, as carbohydrate levels were nearly exhausted by this point (Table 2; Figs. 2 and 3).

**Fig. 4:**
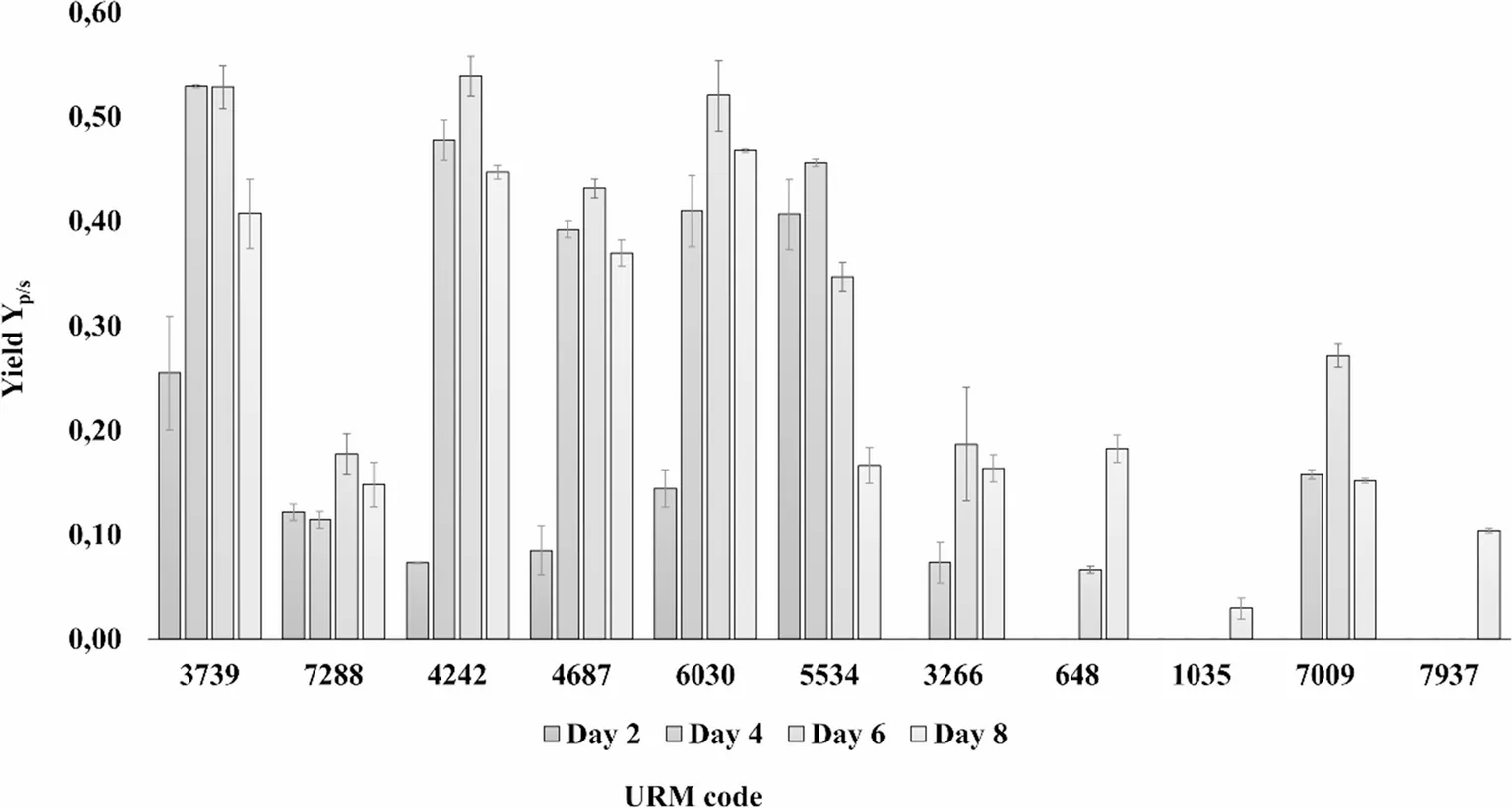
Kojic acid production yields at each fermentation time point by the URM strains of *Aspergillus* section *Flavi* deposited at URM culture collection (Recife, Brazil). *Aspergillus flavus* URM 3739; *A. saccharicola* URM 7288; *A. caelatus* URM 4242; *A. A. luteovirescens* URM 4687; *A. nomiae* URM 6030; *A. parasiticus* URM 5534; *A. tamarii* 3266; *A. novoparasiticus* URM 648; *A. minisclerotigenes* URM 1035; *A. pseudocaelatus* URM 7009 and *A. pseudonomiae* URM 7937

Yields varied over time, with *A. parasiticus* URM 5534 (0.43 ± 0.0 g/g), *A. flavus* URM 3739 (0.22 ± 0.0 g/g), and *A. nomiae* URM 6030 (0.16 ± 0.0 g/g) showing higher yields on the second day of incubation. On the fourth day, *A. flavus* URM 3739 (0.53 ± 0.0 g/g), *A. caelatus* URM 4242 (0.49 ± 0.0 g/g), and *A. parasiticus* URM 5534 (0.46 ± 0.0 g/g) showed the highest efficiency. On the sixth day, the maximum yields were recorded for *A. flavus* URM 3739 (0.54 ± 0.0 g/g), *A. caelatus* URM 4242 (0.53 ± 0.0 g/g), and *A. nomiae* URM 6030 (0.50 ± 0.0 g/g). Finally, on the eighth day, *A. nomiae* URM 6030 (0.47 ± 0.0 g/g), *A. caelatus* URM 4242 (0.45 ± 0.0 g/g), and *A. flavus* URM 3739 (0.41 ± 0.0 g/g) maintained high yield levels (Fig. 4).

## Discussion

The identification of *Aspergillus* species has historically been based on phenotypic characteristics (Thom and Raper 1945; Raper and Fennell 1969; Domsch et al. 1980; Klich and Pitt 1988; Klich 2002). Although these systems were useful at the time, they had limitations in separating morphologically similar species. With the evolution of species concepts, molecular tools have become essential alongside phenotypic analyses for accurate classification.

The majority of strains in this study were deposited in the URM culture collection before the publication of the section *Flavi* review by Varga et al. (2011), and their identification was based mainly on phenotypic characteristics. In our study, the reidentification of the strains was based on *BenA* and *CaM* sequence data. The *RPB2* region, although informative, was difficult to amplify and was not used for the reidentification of previously deposited strains. Molecular data (*BenA* and *CaM*) allowed the recognition of distinct lineages, highlighting their usefulness for species delimitation in *Aspergillus* section *Flavi*.

After the phylogenetic analyses of the 194 strains confirmed as belonging to *Aspergillus* section *Flavi* and stored in the URM culture collection (Recife, Brazil), 11 species were identified and classified into nine series: *Alliacei*, *Annui*, *Avenacei*, *Bertholletiarum*, *Coremiiformes*, *Flavi*, *Kitamyces*, *Leporum*, and *Nomiarum*. This approach was fundamental for understanding species diversity in this group and provided more accurate results for their taxonomic classification. The ITS region was included as the fungal barcode because it is highly conserved in *Aspergillus* and other fungal genera (Samson et al. 2014). However, the ITS region alone is insufficient to separate *Aspergillus* species, requiring additional complementary regions for reliable species distinction (Frisvad et al. 2019; Houbraken et al. 2020). Therefore, *BenA* and *CaM* regions are frequently used for efficient separation of *Aspergillus* species, with *CaM* regarded as the main secondary marker for *Aspergillus* due to its more complete database and ease of amplification (Samson et al. 2014).

However, it was not possible to distinguish between *A. flavus* and *A. oryzae*, or between *A. parasiticus* and *A. sojae*. This result was expected, as previous studies have shown that *A. oryzae* and *A. sojae* are domesticated lineages of *A. flavus* and *A. parasiticus*, respectively (Frisvad et al. 2019; Houbraken et al. 2020). Some researchers report that *A. flavus*, *A. oryzae*, *A. parasiticus*, and *A. sojae* are still considered distinct species mainly for economic and food security reasons, rather than solely on taxonomic differences (Silva et al. 2022a, b). In addition, the isolates identified as *A. arachidicola* (URM 3499 and URM 2581) exhibited distinctive characteristics warranting further investigation; future studies are needed to better understand their taxonomic and biotechnological significance.

Strains identified as *Aspergillus luteovirescens* were isolated from a variety of substrates. Notably, three cultures were recovered from human necropsy samples: two from lung tissue (URM 5985 and URM 5987) and one from brain tissue (URM 6029). To our knowledge, this is the first report of *A. luteovirescens* being isolated from human clinical specimens, suggesting a potential pathogenic role. While this species had not previously been associated with human disease, other members of the series *Nomiarum*, such as *Aspergillus nomiae*, have been implicated in cases of onychomycosis (Zotti et al. 2011), pneumonia (Caira et al. 2012), and keratitis (Manikandan 2009). This finding raises the possibility that *A. luteovirescens* may also act as an opportunistic human pathogen and highlights the importance of further taxonomic studies, particularly re-evaluating clinical isolates previously identified as *A. nomiae* using a robust polyphasic approach to confirm species-level identification.

*Aspergillus minisclerotigenes* (URM 1035 and URM 5791) is reported here for the first time in Brazil, isolated from the substrate ‘castor cake’. *Aspergillus saccharicola* (URM 7288 and URM 2814) is also reported for the first time in soil and for the second time in Brazil, with URM 2814 isolated from sugarcane bagasse and URM 7288 from soil. These findings are particularly significant, as they represent the first records of *A*. *saccharicola* in Northeast Brazil and expand the known geographic and ecological ranges of both species.

*Aspergillus* is known for having species that produce a wide range of secondary metabolites and enzymes, including kojic acid, which has important applications in several industrial sectors. For example, in the pharmaceutical industry, kojic acid is used in skin-lightening products for its melanin-inhibiting properties, while in the food industry, it serves as an antioxidant to prevent oxidative damage and extend shelf life. Although many *Aspergillus* species have been reported as producers or potential producers of kojic acid, mainly those belonging to the *Aspergillus* section *Flavi* (Abd El-Aziz 2013; El-Kady et al. 2014; Frisvad et al. 2019; Rasmey and Abdel-Kareem 2021; Silva et al. 2022a, b; Felipe et al. 2023), in our study, *A. arachidicola*, *A. nomiae*, *A. caelatus*, *A. novoparasiticus*, *A. minisclerotigenes*, *A. pseudocaelatus*, *A. pseudonomiae*, and *A. saccharicola* were evaluated for the first time regarding kojic acid production in submerged fermentation. We used sugarcane molasses and corn steep liquor as the main sources of carbon and nitrogen. The goal was to test whether agricultural waste could be used as a substitute for the traditional medium, which contains expensive ingredients such as glucose, yeast extract, KH₂PO₄, and MgSO₄·7 H₂O (Chib et al. 2019). These alternative substrates showed potential for producing kojic acid cost-effectively and sustainably. The potential for kojic acid production by *Aspergillus* species contributes to the identification of valuable resources and opens new possibilities for developing innovative biotechnological products and processes.

Our study showed that all the URM *Aspergillus* strains evaluated for kojic acid production potential were able to utilize sugarcane molasses and corn steep liquor as their main sources of carbon and nitrogen, both for basal metabolism and for kojic acid synthesis, presenting different growth rates and kojic acid concentrations. These differences may be attributed to strain conditions or species traits (Abd El-Aziz 2013). The carbon source is one of the main components for kojic acid production by fungi. The carbohydrate-rich composition of the alternative medium may have induced acid production by the evaluated *Aspergillus* strains. However, the absence of production or production of quantities below detectable levels indicates the need to optimize the medium and experimental conditions. Despite some discrepancies in the results, under optimized conditions, Petri dishes are considered advantageous due to ease of visualization and identification, and they are applicable to a large number of samples (Olicón-Hernández et al. 2022).

These results are consistent with previous studies that evaluated kojic acid production using sugarcane molasses. For example, Zohri et al. 2018 reported production of 24.65 g/L kojic acid by *A. flavus* isolate No. 3. Based on fermentation times, it can be suggested that kojic acid synthesis occurred after the fungal growth reached the stationary phase and ceased when carbon sources in the medium were depleted or nearly exhausted. Similar behavior was reported in other studies (Rosfarizan et al. 1998; Futamura et al. 2001).

Kojic acid production observed in the URM strains aligns with findings from various studies utilizing different carbon and nitrogen sources. For example, El-Kady et al. (2014) evaluated different strains of *A. flavus* using sugarcane molasses as a carbon source and obtained kojic acid production ranging from 3.7 to 9.3 g/L. Similarly, Zohri et al. (2018) assessed kojic acid production by three *A. flavus* isolates under submerged cultivation using glucose and yeast extract as carbon and nitrogen sources, yielding kojic acid concentrations of 8.5–10.6 g/L. These researchers also achieved a kojic acid concentration of 15.71 g/L using sugarcane molasses as the carbon source. Additionally, Ibrahim and Saleh (2018) evaluated various fungal species, including *A. oryzae*, and obtained 8.01 g/L of kojic acid in PDA medium containing dextrose as the main carbon source.

In this study, yields ranged from 0.02 to 0.53 ± 0.0 g/g. Abd El-Aziz (2013) reported yields between 0.1 and 0.50 g/g; Hazzaa et al. (2013) between 0.002 and 0.447 g/g; and Yan et al. (2014) between 0.31 and 0.36 g/g. The similarity between the yields obtained by the URM strains and those reported in other studies suggests that, with optimized cultivation conditions, URM *Aspergillus* strains have the potential to produce higher concentrations of kojic acid, especially considering that other studies have greater yields under optimized conditions.

*Aspergillus caelatus* URM 4242 was the strain that showed the highest kojic acid production and yield, with 10.65 ± 0.4 g/L and 0.53 ± 0.0 g/g, respectively. The lack of prior studies evaluating kojic acid production by this species under submerged cultivation underscores the need for more detailed research to assess its biotechnological potential. A positive aspect, as noted by Frisvad et al. (2019), is that strains of *A. caelatus* do not produce aflatoxins. Future studies could evaluate *A. caelatus* URM 4242 for mycotoxin production and safety in kojic acid synthesis, particularly for food-industry applications.

Regarding kojic acid production by these strains, it is important to note that kojic acid concentration declined on the final day of fermentation. This reduction is likely related to the depletion of carbon sources in the fermentation medium. Furthermore, kojic acid production during the first two days was significantly reduced, which may be associated with the initial fungal growth phase (El-Aasar 2006). The decrease in kojic acid concentration on day eight may be attributed to its degradation by the mycelium into oxalic and acetic acids, particularly under glucose-depleted conditions (Mohamad and Ariff 2007). Previous studies have also reported this degradation under similar conditions (Bajpai et al. 1981; El-Aasar 2006; Mohamad and Ariff 2007).

The taxonomic re-identification of strains in culture collections plays a crucial role in accurately identifying stored microorganisms, ensuring the reliability of information accessible to the scientific community and industry. The results obtained in this study are considered promising, given that the URM strains are wild fungi preserved over a long period, with the oldest, *A. novoparasiticus* URM 648, deposited in 1954, and the most recent, *A. flavus* URM 8537, in 2021. Remarkably, even after such extended preservation, the strains were still able to produce kojic acid.

The use of sugarcane molasses and corn steep liquor as sources of carbon and nitrogen for kojic acid production proved to be viable and advantageous, due to the low cost and wide availability of these raw materials—particularly in Brazil, a major global producer of sugarcane and corn (Faostat 2021). Moreover, the production of kojic acid, a high-value-added compound, represents a promising application of industrial byproducts such as sugarcane molasses and corn steep liquor, underscoring their potential in sustainable, economically beneficial biotechnological processes. Given global concerns about the production costs of kojic acid—primarily due to its reliance on glucose as the primary carbon source—and the growing market demand for this compound, studies aimed at enhancing its production using low-cost media are highly valuable. Optimizing production processes and developing hyper-producing strains could increase accessibility and competitiveness in the global market.

## Conclusion

This study highlights the importance of culture collections in preserving fungal biodiversity and ensuring the long-term availability of well-documented strains for scientific and biotechnological applications. The results demonstrate that strains stored in the URM culture collection retain their ability to produce kojic acid even after extended periods of preservation. Furthermore, the tested strains efficiently produced kojic acid from low-cost substrates such as sugarcane molasses and corn steep liquor, thereby offering promising opportunities to meet increasing market demand, stimulate industrial growth, and promote the sustainable use of natural resources. This approach also has the potential to make kojic acid more accessible and globally competitive, thereby contributing to significant advances in the biotechnology sector and offering efficient, eco-friendly solutions for the future. In addition, the taxonomic re-identification of the strains improves the reliability of culture collection data and supports more accurate biotechnological exploitation. Overall, these findings emphasize the potential of preserved fungal resources for the development of cost-effective and sustainable bioprocesses.

## Supplementary Information

Below is the link to the electronic supplementary material.

Supplementary Material 1 (DOCX 4.16 MB)

## Data Availability

All data supporting the findings of this study are available within the paper and its Supplementary Information. Additional information will be provided upon request.
