# Supplementary material for: Taxonomic re-identification and biotechnological potential of a Brazilian Aspergillus section Flavi collection: exploring kojic acid production
Source: World J Microbiol Biotechnol. 2026 Jul 18;42(8):419. doi: 10.1007/s11274-026-05137-z (PMC13379445; doi:10.1007/s11274-026-05137-z)

**World Journal of Microbiology and Biotechnology**

**Taxonomic re-identification and biotechnological potential of a Brazilian *Aspergillus* section *Flavi* collection: exploring kojic acid production**

Maria Tamara de Caldas Felipe^1#^; Renan do Nascimento Barbosa^1#^; Jadson Diogo Pereira Bezerra^2^; Rafael Barros de Souza^3^; Walter de Paula Pinto Neto^3^; Rayssa Karla Silva^3^; Cristina Maria de Souza-Motta^1^

^1^ Departamento de Micologia Prof. Chaves Batista, Centro Acadêmico do Recife, Universidade Federal de Pernambuco, Av. Prof. Moraes Rego, s/n, Centro de Biociências, Cidade Universitária, Recife, Pernambuco, CEP: 50670-901, Brazil

^2^ Laboratório de Micologia, Departamento de Biociências e Tecnologia, Instituto de Patologia Tropical e Saúde Pública (IPTSP), Universidade Federal de Goiás (UFG). Rua 235, s/n, Setor Universitário, CEP: 74605-050. Goiânia, Goiás, Brazil

^3^ Laboratório de Metabolismo Microbiano, Instituto de Ciências Biológicas, Universidade de Pernambuco, s/n, Santo Amaro, Recife, Pernambuco, CEP: 50100-130, Brasil

# These authors contributed equally to this work.

*Corresponding author: Maria Tamara C. Felipe (mariatcfelipe@gmail.com); Renan do N. Barbosa (renan.barbosa@ufpe.br)

**Supplementary Table 1** Qualitative evaluation of the potential for kojic acid production by strains of different species of *Aspergillus* section *Flavi* deposited in the Micoteca URM collection (WFCC n° 604).

| **Species** | **URM code** | **Kojic acid production** |
| --- | --- | --- |
| *A. minisclerotigenes* | 1035 | **+** |
| *A. minisclerotigenes* | 5791 | **+** |
| *A. novoparasiticus* | 648 | **+** |
| *A. parasiticus* | 753 | **+** |
| *A. parasiticus* | 2579 | **-** |
| *A. parasiticus* | 5533 | **+** |
| *A. parasiticus* | 5534 | **+** |
| *A. parasiticus* | 7308 | **+** |
| *A. tamarii* | 2161 | **-** |
| *A. tamarii* | 2232 | **+** |
| *A. tamarii* | 3266 | **+** |
| *A. tamarii* | 3488 | **-** |
| *A. tamarii* | 4522 | **-** |
| *A. tamarii* | 4634 | **-** |
| *A. tamarii* | 4876 | **-** |
| *A. tamarii* | 4992 | **-** |
| *A. tamarii* | 5244 | **+** |
| *A. tamarii* | 5265 | **+** |
| *A. tamarii* | 5362 | **-** |
| *A. tamarii* | 5564 | **-** |
| *A. tamarii* | 5778 | **-** |
| *A. tamarii* | 6322 | **-** |
| *A. tamarii* | 6435 | - |
| *A. tamarii* | 6594 | - |
| *A. tamarii* | 6599 | - |
| *A. tamarii* | 6718 | - |
| *A. tamarii* | 6751 | - |
| *A. tamarii* | 7194 | - |
| *A. caelatus* | 4242 | + |
| *A. pseudocaelatus* | 421 | + |
| *A. pseudocaelatus* | 4709 | + |
| *A. pseudocaelatus* | 5963 | - |
| *A. pseudocaelatus* | 7009 | + |
| *A. pseudocaelatus* | 7172 | - |
| *A. nomiae* | 1873 | - |
| *A. nomiae* | 1878 | + |
| *A. nomiae* | 5600 | - |
| *A. nomiae* | 5787 | - |
| *A. nomiae* | 5865 | - |
| *A. nomiae* | 6030 | + |
| *A. nomiae* | 6868 | - |
| *A. pseudonomiae* | 5855 | - |
| *A. pseudonomiae* | 7937 | + |
| *A. luteovirescens* | 4687 | + |
| *A. luteovirescens* | 6029 | + |
| *A. luteovirescens* | 5985 | + |
| *A. luteovirescens* | 5987 | - |
| *A. arachidicola* | 2581 | - |
| *A. arachidicola* | 3499 | + |

**Supplementary Table 2** Chemical composition of sugarcane molasse

| **Test** | **Sugarcane molasse** |
| --- | --- |
| Brix | 77.0±0.0 |
| pH (diluted) | 6.00±0.0 |
| Ash (%) | 6.03±0.0 |
| Total sugar (%) * | 39.9±1.6 |
| Sucrose* | 34.5±0.1 |
| Glucose* | 3.21±0.1 |
| Fructose* | 4.20 ±0.1 |
| Humidity (%) | 15.1±0.7 |
| Acidity | 96.0±0.0 |
| Density (g/cm³) | 1.35±0.0 |

*g/100g g/ 100g of sugar cane molasses, mean values ± standard deviation (n=3)

**Supplementary Figure legends**

**Supplementary Fig. 1** Maximum-likelihood tree construed using sequences of *BenA* of species included in *Aspergillus* section *Flavi*. Values for BS-ML ≥60 % are included near nodes. The strains used in this study are shown in bold. The tree was rooted to *Aspergillus muricatus* NRRL 35674.


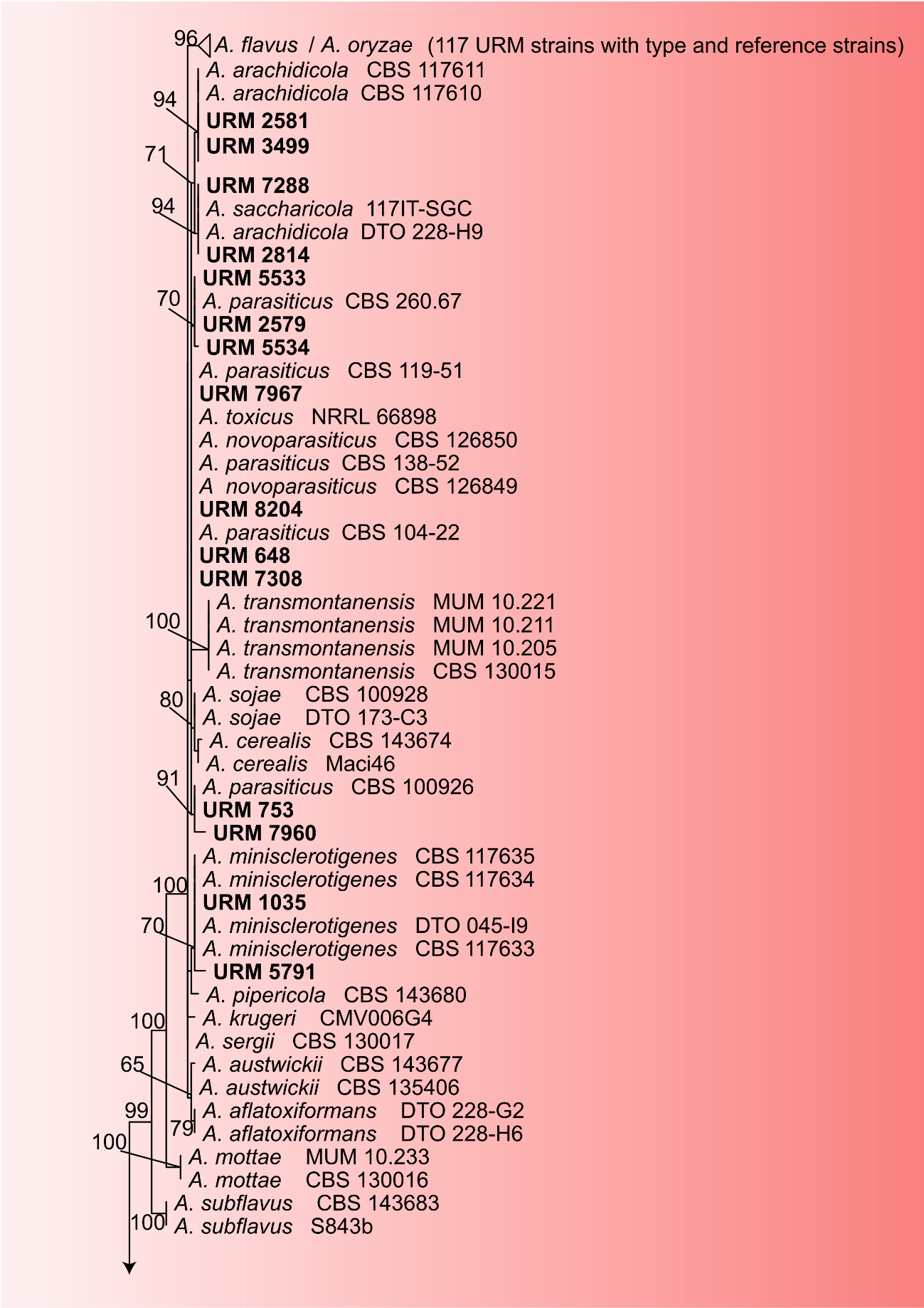


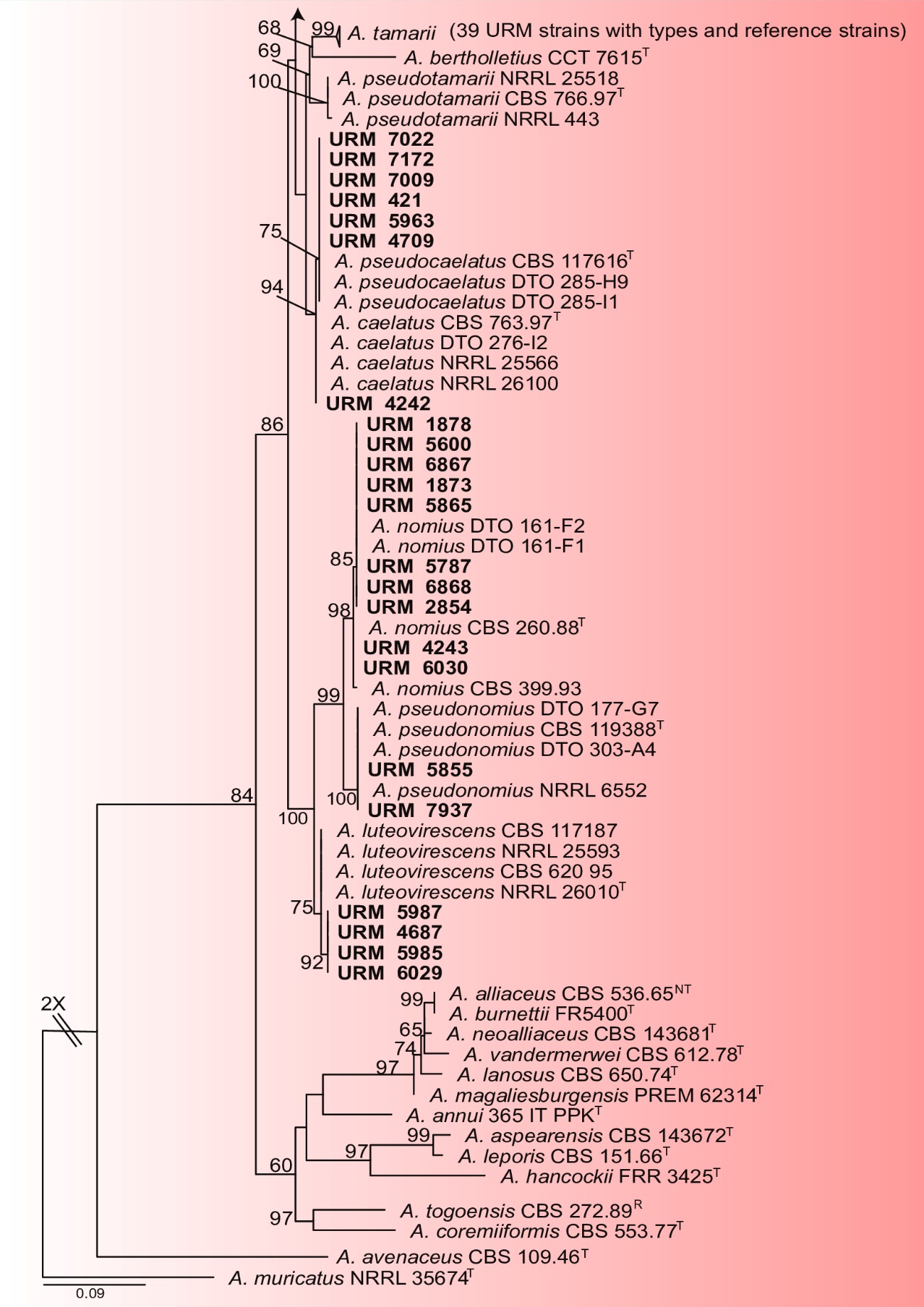


**Supplementary Fig. 2** Maximum-likelihood tree construed using sequences of *CaM* of species included in *Aspergillus* section *Flavi*. Values for BS-ML ≥60 % are included near nodes. The strains used in this study are shown in bold. The tree was rooted to *Aspergillus muricatus* NRRL 35674.


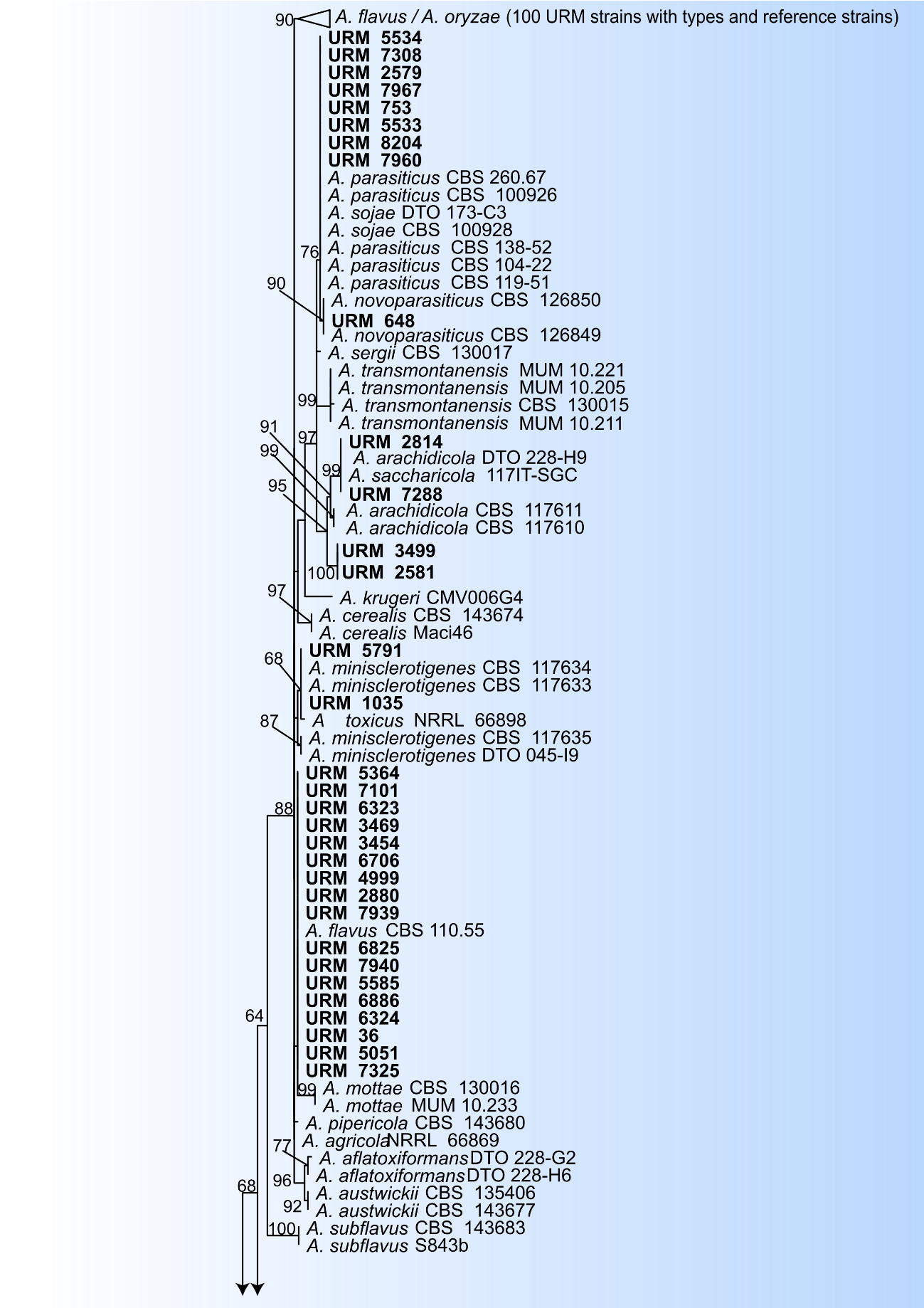


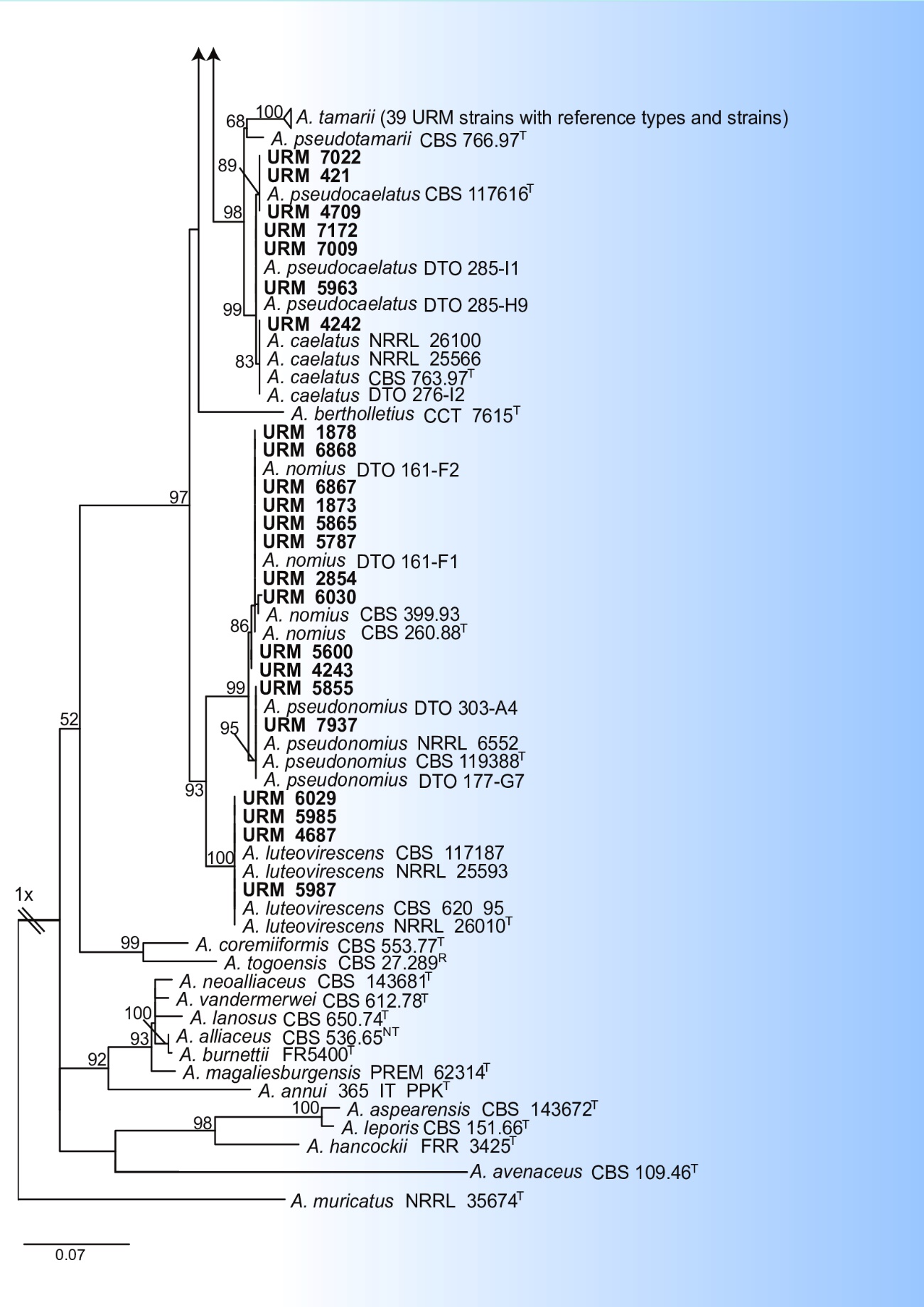


**Supplementary Fig. 3** Representative *Aspergillus* section *Flavi* strains are qualitatively assessed for kojic acid production on solid medium potato dextrose agar (PDA) and sugarcane molasses and corn steep liquor (SMCA). **a** *A. flavus* URM 3739. **b** *A. saccharicola* URM 7288. **c** *A. caelatus* URM 4242. **d** *A. luteovirescens* URM 4687. **e** *A. nomiae* URM 6030. **f** *A. parasiticus* URM 5534. **g** *A. tamarii* URM 3266. **h** *A. novoparasiticus* URM 648. **i** *A. minisclerotigenes* URM 1035. **j** *A. pseudocaelatus* URM 7009. **k** *A. pseudonomiae* URM 7937. **l** *Aspergillus* *arachidicola* URM 3499.


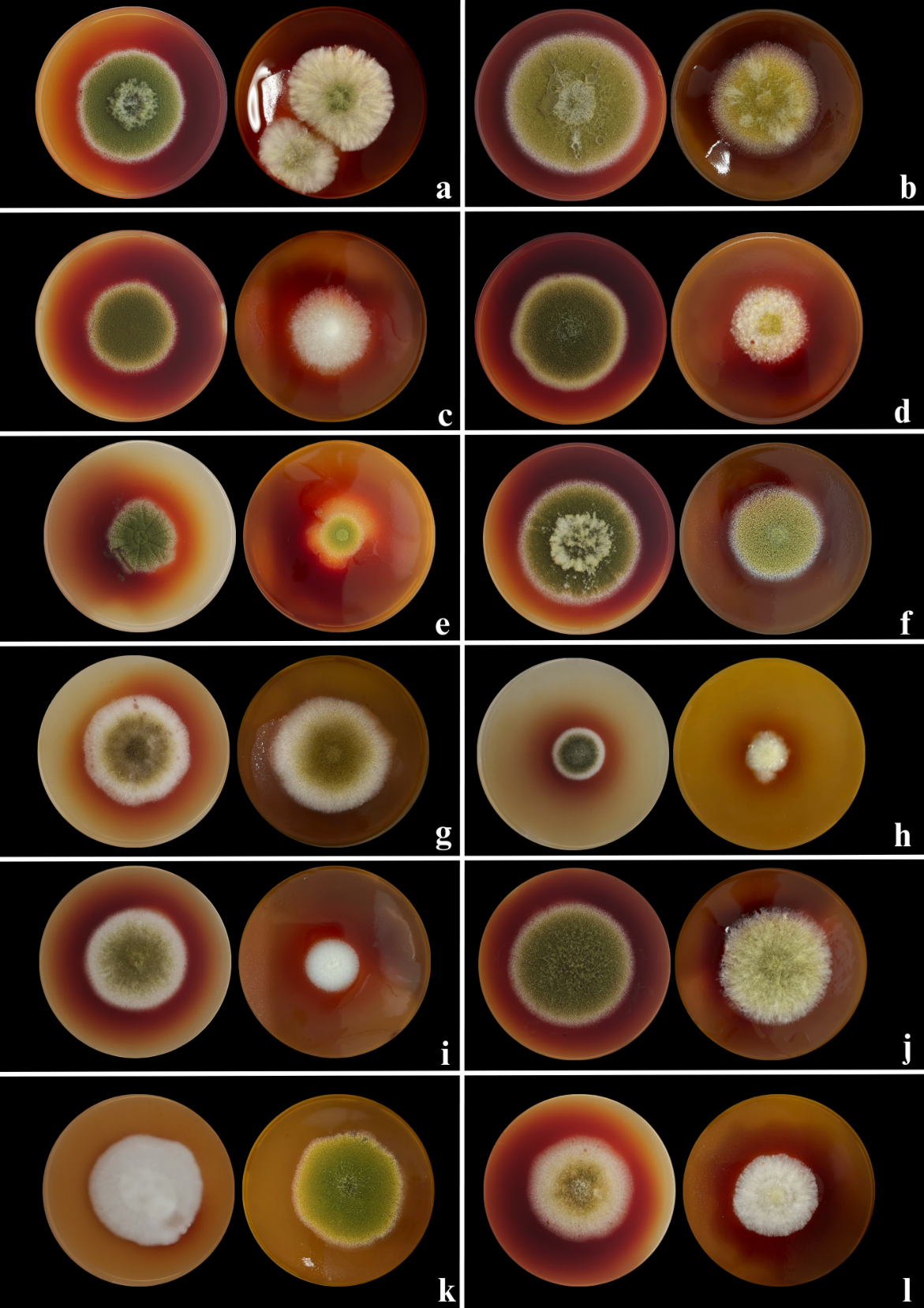

Supplement: Supplementary file 1 — Supplementary Material 1 (DOCX 4.16 MB) [file 11274_2026_5137_MOESM1_ESM.docx]
